# Interplay between androgen and CXCR4 chemokine signaling in myelin repair

**DOI:** 10.1186/s40478-024-01730-1

**Published:** 2024-01-30

**Authors:** Narimène Asbelaoui, Charly Abi-Ghanem, Géraldine Schlecht-Louf, Hania Oukil, Cindy Degerny, The Netherlands Brain Bank, Michael Schumacher, Abdel Mouman Ghoumari

**Affiliations:** 1 UMR1195, “Diseases and Hormones of the Nervous System”, Inserm and University Paris-Saclay, 80, Rue du Général Leclerc, 94276 Kremlin-Bicêtre, France; 2 INSERM UMR 996, Inserm, Inflammation, Microbiome and Immunosurveillance, Faculté de Pharmacie, Université Paris-Saclay, Orsay, France; 3 Netherlands Institute for Neuroscience, Amsterdam, The Netherlands; 4 Present Address: Department of Neuroscience and Experimental Therapeutics, Albany Medical College, Albany, NY 12208 USA

**Keywords:** Remyelination, Androgen receptor, Testosterone, Chemokine receptor, Astrocytes, Schwann cells

## Abstract

In men, reduced levels of testosterone are associated with the prevalence and progression of multiple sclerosis (MS), a chronic and disabling demyelinating disorder. Testosterone has been shown to promote myelin repair. Here, we demonstrate that the cooperation between testosterone and CXCR4 signaling involving astrocytes is required for myelin regeneration after focal demyelination produced in the ventral mouse spinal cord by the infusion of lysolecithin. The testosterone-dependent remyelination of axons by oligodendrocytes was accompanied by an increase in astrocytes expressing CXCR4, its ligand CXCL12 and the androgen receptor (AR) within the demyelinated area. Depriving males of their testosterone or pharmacological inhibition of CXCR4, with the selective antagonist AMD3100, prevented the appearance of astrocytes expressing CXCR4, CXCL12 and AR within the demyelinated area and the concomitant recruitment of myelin forming oligodendrocytes. Conditional genetic ablation of either CXCR4 or AR in astrocytes also completely blocked the formation of new myelin by oligodendrocytes. Interestingly, the gain of function mutation in CXCR4 causing WHIM syndrome allows remyelination to take place, even in the absence of testosterone, but its potentiating effects remained observable. After testosterone deprivation or CXCR4 inhibition, the absence of astrocytes within the demyelinated area led to the incursion of Schwann cells, most likely derived from spinal nerves, and the formation of peripheral nerve type myelin. In patients with progressive MS, astrocytes expressing CXCR4 and AR surrounded myelin lesions, and their presence opposed the incursion of Schwann cells. These results highlight a mechanism of promyelinating testosterone signaling and the importance of normalizing its levels in combined myelin repair therapies.

**Supplementary Information:**

The online version contains supplementary material available at 10.1186/s40478-024-01730-1.

## Introduction

In the central nervous system (CNS), oligodendrocytes form insulating myelin sheaths around axons, ensuring rapid electrical communication between neurons and providing trophic support [1]. Damaged myelin can be partly replaced by spared oligodendrocytes and the recruitment of oligodendrocyte progenitor cells (OPC), followed by their differentiation into myelinating oligodendrocytes [2]. As remyelination often remains insufficient and fails in chronic lesions, enhancing its regenerative capacity has become a therapeutic option for multiple sclerosis (MS) [3]. However, trials aimed at promoting myelin repair have so far not translated into the clinics [4].

The often-neglected hormonal status of MS patients warrants particular attention for efficient remyelination strategies. In men with MS, low levels of testosterone (T) resulting from dysfunctions of the hypothalamus and pituitary gland, have indeed been associated with neurological disability and worse clinical outcomes [5, 6]. Moreover, the risk of hospital admission for MS was significantly increased in men diagnosed for testicular hypofunction [7]. Correspondingly, in the most commonly used animal model to investigate MS pathology, in experimental autoimmune encephalomyelitis (EAE), levels of T in brain and spinal cord were markedly reduced [8, 9]. Importantly, treatment with T ameliorated disease symptoms in EAE mice [10–12].

We have previously shown that treatment with T stimulated the formation of new myelin and reversed myelin damage in a mouse model of chronic demyelination [13]. Moreover, the spontaneous regeneration of myelin by oligodendrocytes was compromised after castration and restored by T treatment after focal lesion of ventral spinal cord or corpus callosum myelin by lysolecithin (LPC) infusion [11, 14]. Conditional ablation of the androgen receptor (AR) within the CNS abolished T-dependent remyelination, identifying the receptor as a drug target for remyelination [14].

In this study, we demonstrate that T increases expression of the chemokine receptor CXCR4 and the appearance of astrocytes expressing CXCR4 and its chemokine ligand CXCL12 after LPC demyelination. CXCL12/CXCR4 signaling has been shown to regulate OPC proliferation, migration and their differentiation into myelin-forming oligodendrocytes [15, 16] and has been proposed as a target for promoting remyelination [17]. Importantly, astrocytes within the area of demyelination also express AR, and we demonstrate that T and CXCR4 signaling are interdependent in stimulating myelin regeneration by oligodendrocytes. The appearance of CXCL12/CXCR4 and AR expressing astrocytes within the demyelinating lesion is a prerequisite for oligodendrocyte remyelination, as in their absence, axons were instead remyelinated by Schwann cells, most likely originating from spinal nerve roots.

Noteworthy are the almost all-or-nothing effects of the inhibition of either T or CXCR4 signaling in preventing the recovery of astrocytes and oligodendrocyte remyelination. These experimental findings may be relevant for MS. We indeed observed that astrocytes surrounding areas of myelin loss on spinal cord sections of MS patients, the so-called plaques, coexpressed CXCR4 and AR, but never colocalized with Schwann cells. The prerequisite of interactive T and CXCR4 signaling for oligodendrocyte remyelination provides support for effective remyelination therapies based on the restoration of normal androgen levels.

## Materials and methods

### Animals

Mice were housed in standard plastic cages with 1–5 littermates in a 12-h/12-h light/dark cycle in a temperature-controlled room (~ 21 °C), with ad libitum access to food and water. The care and use of mice were conformed to institutional policies and guidelines (INSERM, French and European Community council directive 86/806/EEC). All mice were between 8 and 12 weeks old and were bred under the C57BL6/J background.

Males were castrated and females were ovariectomized about 2 weeks prior to LPC lesion. The very low levels of testosterone, probably of adrenal origin, were similar in the gonadectomized males (0.21 ± 0.03 nM) and females (0.22 ± 0.06 nM) as determined by GC/MS. Levels 5α-dihydrotestosterone (5α-DHT) and estradiol were below detection limit for both sexes (0.3 nM for 5α-DHT and 20 pM for estradiol).

AR^NesCre^ mice were used. In contrast to other Nestin-Cre mice, this mouse line selectively expresses the Cre in neuronal and macroglial cell precursors, microglial cells being spared [18, 19]. This Nestin-Cre mouse has been used to completely inactivate the AR in the CNS by crossing with floxed AR mice (ARfl/Y). Both strains were on a C57/BL6 background [13, 14, 20].

PLP-eGFP mice expressing the enhanced green fluorescent protein (eGFP) in cells of the oligodendroglial lineage under the control of PLP gene promoter were obtained from Wendy Macklin (University of Colorado, Aurora, CO) [21].

We created CXCR4^GFAPCre^ mice with selective ablation of CXCR4 in astrocytes by crossing Cxcr4tm2Yzo/J mice (exon 2 of the CXCR4 gene flanked by LoxP sites, CXCR4^Lox/Lox^ mice, RRID:IMSR_JAX:00876) with GFAP^Cre^ mice line 77.6 (Cre recombinase under the control of the GFAP gene promoter, RRID:IMSR_JAX:024098). In contrast to other GFAP^Cre^ mice, the GFAP^Cre^ line 77.6 targets astrocytes throughout the healthy and injured brain and spinal cord, with only additional Cre expression in a small population of cells within the subependymal zone of the adult brain [22, 23]. Both lines were purchased from Jackson Laboratory (Bar Harbor, ME, USA). They were maintained on a C57BL/6J background. We first generated double heterozygous mice for GFAP^Cre^ and floxed CXCR4 (GFAP^Cre^;CXCR4^lox/+^) that were then bred to CXCR4^Lox/Lox^ mice via a back-mating strategy to generate excised floxed CXCR4 homozygous (GFAP^Cre^;CXCR4^Lox/Lox^), that we called CXCR4^GFAPCre^, and heterozygous (GFAP^Cre^;CXCR4^lox/+^) for floxed CXCR4. Cre-null mice (CXCR4^lox/+^ and CXCR4^lox/lox^) were used as controls throughout this study. No special phenotype has been noticed. For genotyping, genomic DNA was isolated from tail tips using the DNeasy Blood and Tissue kit (ID: 69504, Qiagen). The same procedure was applied to produce AR^GFAPCre^ mice (mice lacking AR in their astrocytes) by mating GFAP^Cre^ mice line 77.6 to AR^Lox/Lox^ mice.

Finally, adult male Cxcr4^+/1013^ mice harboring the CXCR4^1013^ gain of function mutation on the C57BL/6J background were used [24]. This mutation causes the WHIM “Hypogammaglobulinemia, Infections, and Myelokathexis” syndrome [25]. In the mouse strain we used, the gain of CXCR4 function mutation is not restricted to astrocytes and affects all CXCR4-expressing cells, promoting their migration towards CXCL12 [24, 25].

### Focal demyelination with lysolecithin (LPC)

Demyelination was induced by injection of 1% LPC (lysophosphatidylcholine, Sigma-Aldrich) in phosphate buffered saline (PBS) into the right ventral funiculus of 2–3 months old castrated male or ovariectomized female mice anesthetized with ketamine (80 mg/kg) and xylazine (10 mg/kg).

LPC was injected bilaterally at 0.6 mm lateral to the midline of the spinal cord and at 1.43 to 1.46 mm depths between T11 and T12. The central vein was used as a benchmark. Injection was performed with glass capillaries (diameters not exceeding 50 µm) connected to a Hamilton syringe controlled by an infusion pump delivering 0.1 µl/min during 10 min. Under these experimental conditions, the staining of large-caliber axons with an antibody against neurofilament 200 kDa (NF-200) revealed no differences after LPC-induced demyelination between animals receiving an empty or a T-filled s.c. implant at 30 dpl [14]. It is worth to be mentioned that treatment with LPC did not produce significative changes in steroid levels of intact males, most likely because the focal lesion localized to the spinal cord.

After LPC infusion, castrated males or ovariectomized females received at the end of the surgical procedure a subcutaneous (s.c.) 10 mm Silastic implant (inner diameter: 1.57 mm; outer diameter: 2.41 mm; Dow Corning), either empty or filled with T (Fluka 86500). Then, AMD3100 (Sigma) were subcutaneously injected every 2 days. Among four different concentrations of AMD3100 tested (0.5, 1, 5 and 10 µM of AMD3100), the optimal doses for males (5 mg/kg) and females (1 mg/kg) was chosen. Mice were sacrificed at 5-, 10-, 15- or 30 dpl for immunohistological analysis. For gene chip and electron microscopy analysis, mice were sacrificed at 5 or 30 dpl.

### Laser capture microdissection

To study the effect of T on gene expressions after LPC-induced demyelination, 2 groups of 4 castrated male mice received and empty or T-filled s.c. Silastic implant. At 5 dpl, mice were sacrificed and frozen spinal cord sections of 10 µm were cut. The lesion was visualized with Cresyl violet. Regions of interest were microdissected using a microscope coupled to an infrared laser (Arcturus XT™ Laser Capture Microdissection System, Applied Biosystems). Microdissection was performed at the Plateforme HistIM at Institut Cochin (Paris). Selected regions were collected onto specific caps (CapSure Macro LCM Caps, Applied Biosystems), and RNAs were extracted (RNeasy Micro Kit (74004, Qiagen).

### Affymetrix chip data analysis

After, quantification and quality evaluation, RNAs were hybridized to Affymetrix chip "mouse gene 2.1 ST" to analyze differentially expressed gene. Those presenting a fold change superior of 2 were selected. Data analysis of differentially expressed genes were conducted using the Partek Genomic Suite software (Partek Inc., St Louis, MO, USA), in collaboration with the Translational Research Department, Genomics Platform of the Institut Curie (PSL Research University, Paris).

### Real time PCR

In order to confirm the differential expression of CXCR4 and MPZ (P0) genes between T and vehicle treatments, we performed a quantitative real-time PCR (qRT-PCR) on the same RNA used for Affymetrix chip analysis. We also extracted total RNA from brains of castrated adult males treated with PBS instead of LPC or PBS + T and from brains of (AR^lox/lox^ and AR^NesCre^ mice) and purified them using RNeasy Purification Reagent (Qiagen). To obtain cDNA, reverse transcriptase was carried out using 10 µg of total RNA. qRT-PCR was performed using SYBR Green PCR Master Mix (Applied Biosystems, Foster City, CA, USA). The primers used for targeting CXCR4 and MPZ genes are. For CXCR4 gene (Reverse 5‘-GAT GGG ATT TCT GTA TGA GGA TTA-3’; Forward 5‘-CCA CCC AGG ACA GTG TGA CTC TAA-3’) and for MPZ gene (Reverse 5‘-AGACTACAGTGACAACGGCA-3’; Forward 5‘-AGAAGAGCAACAGCAGCAAC-3’). Relative expression of these genes has been determined by using the Eq. 2-ΔΔCt and was reported to the house keeping gene Cyclophilin A.

### Organotypic cerebellar and spinal cord slice cultures

Cultures of 350 µm thick cerebellar slices were prepared from P10 PLP-eGFP pups as described earlier [13, 26]. Slices were cultured on Millipore membranes of 30 mm diameter with a 0.4 µm pore size (Millicell, Millipore) at 37 °C in a 5% carbon dioxide (CO_2_) humidified atmosphere. The medium was changed every 2 to 3 days. To induce demyelination after 7 days in culture, 0.5 mg/ml LPC (Sigma) was added fresh medium for 17–18 h. After removing the medium, slices were incubated for 5 additional days in the absence or presence of T (1 µM in 0.1% ethanol) and AMD3100 (5 µM).

### Coculture of organotypic brain slices

After 7 days culture, P10 C57BL6/J cerebellar slices were demyelinated with LPC (0.5 mg/ml during 17–18 h). They were then apposed to portions of brain stem slices from newborn transgenic P0 PLP-eGFP mice (modified from [13]). This coculture system allowed us to study the migration of PLP-eGFP^+^ oligodendroglial cells into LPC-demyelinated slices. The medium was replaced and treatments renewed after 2–3 days. After 10 days, the number of EGFP^+^ cells that had migrated into the demyelinated slice and the distance of their migration from the limit between the two slices were quantified.

### Identification and immunohistochemical analysis of MS lesions

The Netherlands Brain Bank (NBB) provided postmortem materials and clinical data of multiple sclerosis (MS) donors. Donors provided informed consent for brain autopsy and for the use of material and clinical data for research purposes in compliance with national ethical guidelines and the NBB donor program has been approved by the Ethical Committee VU University Medical Center (Amsterdam). The clinical course was defined as primary progressive (PP) or secondary progressive (SP). Disability status was determined by retrospective chart analysis using Kurtzke’s Expanded Disability Status Scale (EDSS). Multiple sclerosis tissue sample identity for each patient and clinicopathologic data are summarized in Additional file 1: Table S2. No significant differences were found in age at death, post-mortem delay, post-mortem pH between sexes. Spinal cord tissue samples at thoracic level from 5 women and 5 men with MS disease were used in this study. They have been received in embedded paraffin and were cut in 5 µm sections before being analyzed by immunolabeling antibodies against MBP, GFAP, CXCR4 and AR.

### Immunohistochemistry

Mice were deeply anaesthetized with a mixture of ketamine-xylazine and then fixed by transcardiac perfusion with a 4% freshly prepared paraformaldehyde solution in PBS (0.1 M, pH 7.5). Extracted spinal cords were post-fixed using 4% paraformaldehyde, included into paraffin blocks from which transversal sections of 5 µm were cut using microtome (Micro HM 340E, ThermoScientific). After deparaffinization, rehydration and epitope retrieval in 1X citrate buffer solution, sections were blocked for 1 h in Sea buffer (Sigma) before immunostaining. Primary antibodies were incubated overnight at 4 °C followed by PBS 1X washing and incubation with secondary antibodies at room temperature for 2 h. The antibodies used in our study are listed in Additional file 1: Table S3. For human spinal cord samples, the same protocols of deparaffinization and immunochemistry process were applied. Ex vivo, cultures and cocultures of cerebellar slices were fixed using PFA 4% to perform immunofluorescence, as previously described [27].

### Semi-thin plastic sections and electron microscopy

After the sacrifice of mice, spinal cords were extracted and put in a mixture of 2% paraformaldehyde and 2% glutaraldehyde (Sigma) for 2 weeks. Post-fixation was then carried out in osmium oxide and embedded in epoxy resin. To define LPC lesions, semi-fine sections of (0.5 µm) were cut and stained with toluidine blue. After visualization of the LPC lesion, ultrathin Sects. (60 nm) were cut and myelin structure was analyzed using transmission electron microscopy (Philips EM 208).

### Immunohistological analysis

Processed spinal cord slices were permanently mounted and pictures were taken using confocal (Zeiss LSM510-Meta) and AxioImager A1 microscopes. For each cerebellar slice culture, three different cerebellar lobules were photographed. For spinal cord sections, the center and border (RIM) of demyelinated areas were photographed. All analysis was performed by NIH Fiji software. To evaluate in vivo the expression of MBP, GFAP, CXCR4 and CXCL12, immunostaining densities were quantified. The quantification of fluorescence was normalized to the lesion area. For all tissues (lesioned and lesioned + T), regions of interest (ROI) were isolated from the rest of the image and areas outside of the ROI were cleared. A threshold was set for the standardization of different measurements and the same threshold intensity range was maintained for all images. Then, the area covered by a positive staining signal (immunolabelled material: gray scale pixels) was calculated as a % of staining within the ROI area by dividing the number of gray scale pixels by the total number of gray and white scale pixels multiplied by 100. White scale pixels correspond to area covered by the background ‘noise’ (non-specific staining). In organotypic slice cultures, lesions are multiple and diffuse, so a surface of 0.26 mm^2^ was designed to calculate the cell number.

### Statistical analysis

The G*Power 3.1.9.2 computer program software was used to perform the calculation of required sample size. The minimal significance (α) and statistical power (1 − β) were set at 0.05 and 0.7, respectively, to detect a difference of 30%. Group sizes were calculated based on our previous studies. To avoid biases in cell quantifications, any cell which did not present marking surrounding the nuclear DAPI or at least largely associated with it was not counted. For the % of immunostaining quantifications, the same and suitable threshold was applied for all images before any measurement. All analyses were conducted by experimenters blinded to the experimental conditions.

Difference between multiple groups were analyzed by two-way or one-way ANOVA followed by Tukey's multiple comparisons tests. Differences between 2 groups were analyzed by two-tailed unpaired Student's t-test (GraphPad Prism, v.8; GraphPad Software, La Jolla, CA, USA, www. graphpad.com). Outliers were identified and rejected using Grubbs’ test. Data are presented as means ± S.E.M and significant differences are marked by asterisks (****P < 0.0001, ***P < 0.001, **P < 0.01, *P < 0.05). The experimenter/analyzer was blinded to experimental conditions.

## Results

### Testosterone-induced upregulation of *CXCR4* and downregulation of *MPZ* gene expression in the lysolecithin model of spinal cord demyelination

**Fig. 1:**
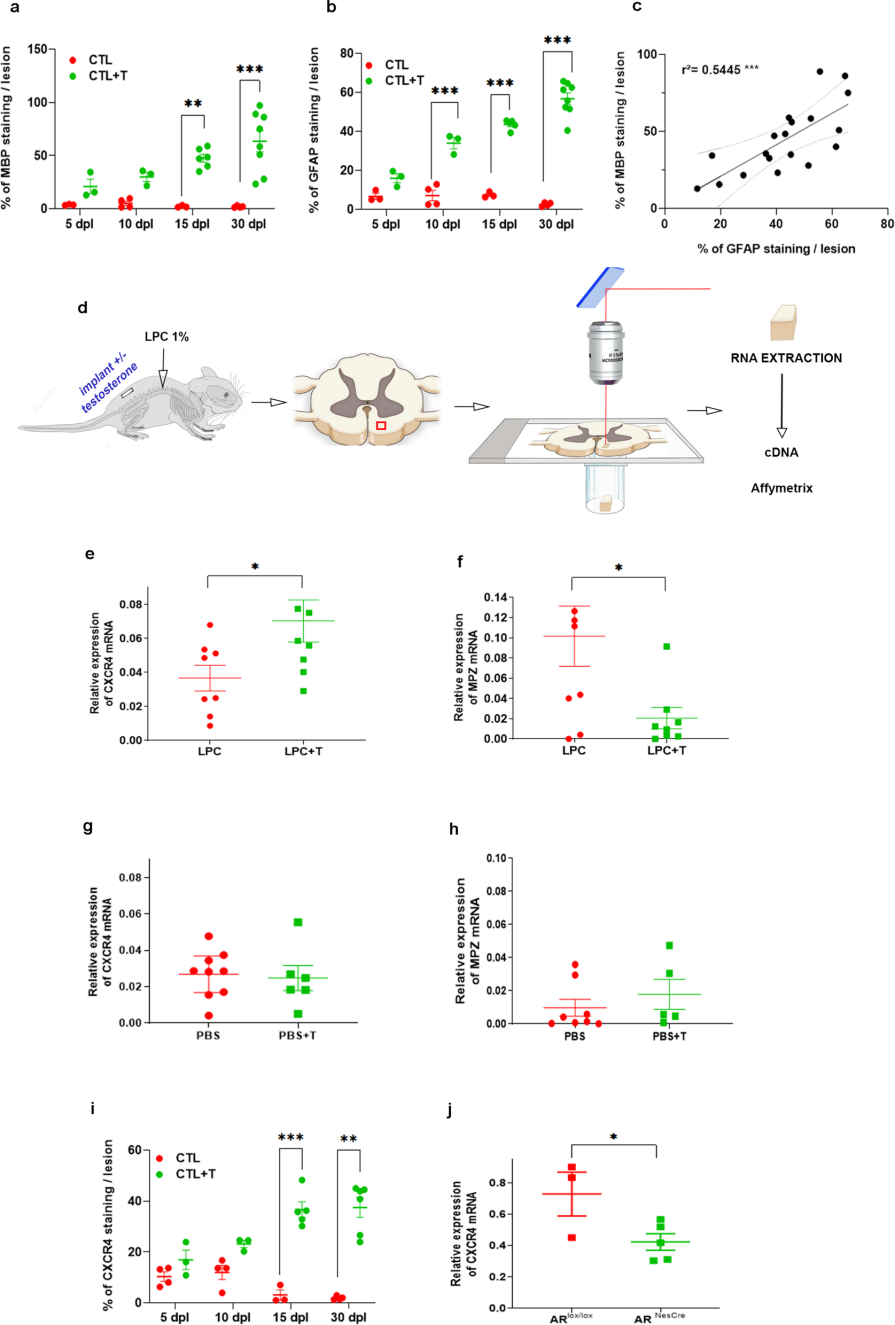
Remyelination, astrocyte replenishment, upregulation of CXCR4 mRNA and downregulation of MPZ mRNA by T in microdissected LPC lesions. **a** and **b** Immunostaining of MBP (**a**) and GFAP (**b**) in the LPC demyelinated lesion at 5, 10, 15 and 30 days post-lesion (dpl). Adult castrated male mice received a subcutaneous (s.c.) Silastic implant, empty (CTL, control) or filled with testosterone (T). CTL indicates LPC and CTL + T indicates LPC + T. Data are presented as means ± S.E.M. (two-way ANOVA with Tukey's multiple comparisons tests). **c** Positive correlation between MBP and GFAP staining within the LPC lesion (Pearson's r = 0.54, ***p < 0.001). **d** LPC-induced demyelination and laser microdissection of the lesion area on spinal cord sections (red square) followed by Affymetrix ChIP analysis. **e** and **f** Relative expression of two candidate genes, CXCR4 (**e**) and MPZ (**f**), assessed by qRT-PCR on RNA from LPC-demyelinated area at 5 dpl. Castrated males were treated with an empty (LPC) or T-filled (LPC + T) implant. Analysis of CXCR4 (**g**) and MPZ (**h**) in uninjured control animals (treated with PBS instead of LPC). Data are presented as means ± S.E.M. (two-tailed unpaired Student's t-test). **i** Immunostaining of CXCR4 in the LPC demyelinated lesion at 5, 10, 15 and 30 dpl. Castrated males were treated with an empty (LPC) or T-filled (LPC + T) implant. Data are presented as means ± S.E.M. (two-way ANOVA with Tukey's multiple comparisons tests). **j** Relative expression of CXCR4, assessed by qRT-PCR on RNA extracted from the spinal cord of AR^Lox/Lox^ and AR.^NesCre^ male mice. Data are presented as means ± S.E.M. (two-tailed unpaired Student's t-test). Asterisks mark significant differences. ***P < 0.001, **P < 0.01, *P < 0.05

Testosterone (T)-dependent myelin regeneration by oligodendrocytes, reflected by immunofluorescence staining of myelin basic protein (MBP), starts as soon as 5 days post-lesion (dpl) and progresses during the first two weeks, becoming dense between 15 and 30 dpl (Fig. 1a). As previously noted, the increase in MBP staining was paralleled by a T-stimulated increase in activated astrocytes immunoreactive for glial fibrillary acidic protein (GFAP) within the demyelinated area (Fig. 1b). There was indeed a significant positive correlation over time between MBP and GFAP immunostaining, pointing to a role of astrocytes in the T-dependent remyelination by oligodendrocytes (Fig. 1c).

To identify genes involved in T-dependent remyelination, we performed RNA profiling within laser capture microdissected lesion areas by Affymetrix microarray analysis at 5 dpl, when the remyelination of axons starts (Fig. 1d). Castrated male mice were treated with an empty or T-filled subcutaneous (s.c.) Silastic implant. The T implant produced levels of T within the normal physiological range of adult males (plasma: 11.9 ± 1.4; brain: 8.3 ± 0.27 nM), as analyzed by gas chromatography/mass spectrometry (GC/MS). Within the tissue, the administered T was partially converted to 5α-DHT (1.72 ± 0.14 nM) and estradiol (190 ± 10 pM).

Hierarchical clustering identified 41 genes with a > twofold fold change (FC) in expression (Additional file 1: Table S1), among them the chemokine receptor CXCR4 and the Schwann cell myelin protein zero (MPZ or P0). In the presence of T, CXCR4 was upregulated (FC =  + 2.11) and MPZ was downregulated (FC =  − 2.57). We focused on these two genes, in particular on CXCR4, which has previously been shown to play a key role in the recruitment of OPC and their differentiation into myelinating oligodendrocytes [15, 16]. MPZ^+^ Schwann cells instead of oligodendrocytes have been reported to remyelinate CNS axons in the absence of T [14].

The microarray results for CXCR4 and MPZ mRNA were first validated by qRT-PCR analysis at 5 days after LPC. At this early time point, treatment of castrated male mice with T significantly upregulated CXCR4 mRNA levels (Fig. 1e) and downregulated MPZ mRNA levels (Fig. 1f). In contrast, in unlesioned animals injected with PBS instead of LPC, treatment with T had no effect on CXCR4 (Fig. 1g) and MPZ (Fig. 1h) mRNA levels, showing that the hormone effect was dependent on the lesion status of the spinal cord tissue. A time-course analysis within the lesion area showed that in the absence of T, CXCR4 immunolabeling remained low during 30 days after LPC, becoming even less intense after 15 days, but progressively increased in T-treated males, reaching highest levels between 15 and 30 dpl, thus paralleling the increase in MBP^+^ myelin (Fig. 1i). The effect of T on CXCR4 expression involved the intracellular AR, as it was downregulated in AR^NesCre^ mice displaying CNS-selective ablation of AR (Fig. 1j) [13, 19, 20], suggesting that CXCR4 acts downstream of AR.

### Testosterone triggers the appearance of astrocytes expressing CXCR4 and its ligand CXCL12 in the demyelinated lesion

**Fig. 2:**
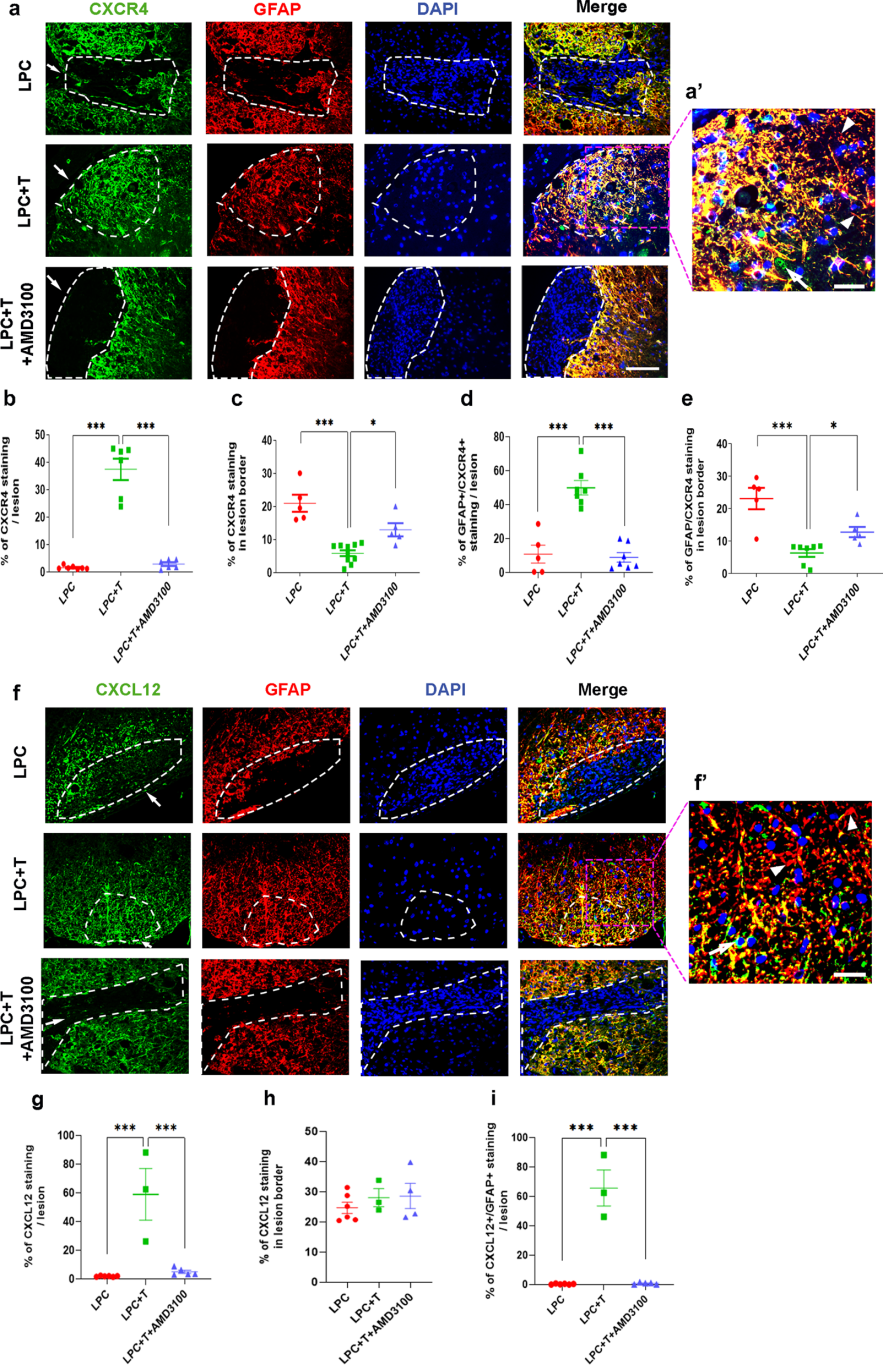
Chemokine receptor CXCR4 and its ligand CXCL12 are expressed by astrocytes. **a**–**d** Double labeling of CXCR4 and GFAP on spinal cord cross sections of castrated males at 30 dpl. Cell nuclei were counterstained in blue with DAPI. Within the LPC lesion, delimited by the dotted line, CXCR4 and GFAP staining were very low in males receiving an empty implant (LPC), but high levels of CXCR4 and GFAP colocalized in males receiving an implant filled with T (LPC + T) (**a**, **b**, **d**). **a′** represents the enlargement of CXCR4/GFAP colabeling under the LPC + T condition. AMD3100 blocked the T-dependent appearance and colocalization of CXCR4 and GFAP inside the lesion. The arrival of CXCR4^+^ astrocytes in the lesion coincided with their reduction at the borders (**c**, **e**). **f**–**i**, Double labeling of CXCL12 and GFAP on spinal cord cross sections of castrated males at 30 dpl. CXCL12 and GFAP staining were very low in control males (LPC), but elevated in males receiving an implant filled with T (LPC + T). **f′** represents the enlargement of CXCL12/GFAP colabeling under the LPC + T condition. AMD3100 blocked the T-dependent appearance and colocalization of CXCL12 and GFAP inside the lesion. The arrival of CXCL12^+^ astrocytes in the lesion was not accompanied by a reduction in CXCL12 staining at the borders (**h**). Arrows indicate no overlapping of CXCR4 and GFAP expression in **a′** and expression of CXCL12 in GFAP^+^ cells bodies in **f′**. Arrow heads indicate no expression of CXCR4 (**a′**) and CXCL12 (**f′**) in the whole astrocyte branching. Data are presented as means ± S.E.M. (one-way ANOVA with Tukey's multiple comparisons tests). Asterisks mark significant differences. ***P < 0.001, *P < 0.05. Scale bars: 20 µm in **a** and **f** and 10 µm in **a′** and **f′**

Analysis of CXCR4 expression by immunohistochemistry at 30 dpl showed very little staining within the lesion area in castrated males receiving an empty implant, but a high density of immunoreactive cells was observed in T-treated males (Fig. 2a, b). In response to T, CXCR4^+^ cells filled the demyelinated lesion, as suggested by the reduction in the dense CXCR4 immunostaining at the lesion borders concomitant with its appearance inside the lesion (Fig. 2a–c). Treatment with AMD3100, a specific inhibitor of CXCR4, blocked the T-dependent appearance of CXCR4^+^ cells within the lesion, which stayed around its borders as in the absence of T (Fig. 2a–c). Double immunostaining revealed that the CXCR4^+^ cells that replenish the lesion were GFAP^+^ astrocytes (Fig. 2a, a′ and d).

The CXCR4 dependency of the effect of T on the replenishment of the LPC lesion by astrocytes suggested the presence of CXCL12, its unique chemokine ligand. Double-immunolabeling indeed showed that GFAP^+^ astrocytes within the lesion area of T-treated males also expressed CXCL12, indicating autocrine chemokine signaling (Fig. 2f–i). As for CXCR4^+^ astrocytes, the appearance of CXCL12^+^ astrocytes inside the lesion was blocked by AMD3100, but it was not accompanied by a significant decrease in CXCL12 around the lesion (Fig. 2h).

The density of DAPI^+^ cells within the lesion was reduced in response to T treatment at 30 dpl, which could reflect a T-dependent reduction in infiltrating immune cells. However, the density of Iba-1^+^ microglial cells was increased by T within the lesion (LPC: 5%, LPC + T: 22% and LPC + T + AMD3100: 3% Iba-1 staining/lesion). An increase in Iba-1 ^+^ microglia after T treatment was also observed at an earlier time point (10 dpl; Additional file 1: Fig. S1). In the absence of T, a reduced density of microglial cells may result in the insufficient phagocytosis of myelin and cell debris. Schwann cells invading the lesion may contribute to the elevated density of DAPI staining observed in the absence of T or after treatment with AMD3100 (see below).

### Astrocytes and neurons express the androgen receptor

**Fig. 3:**
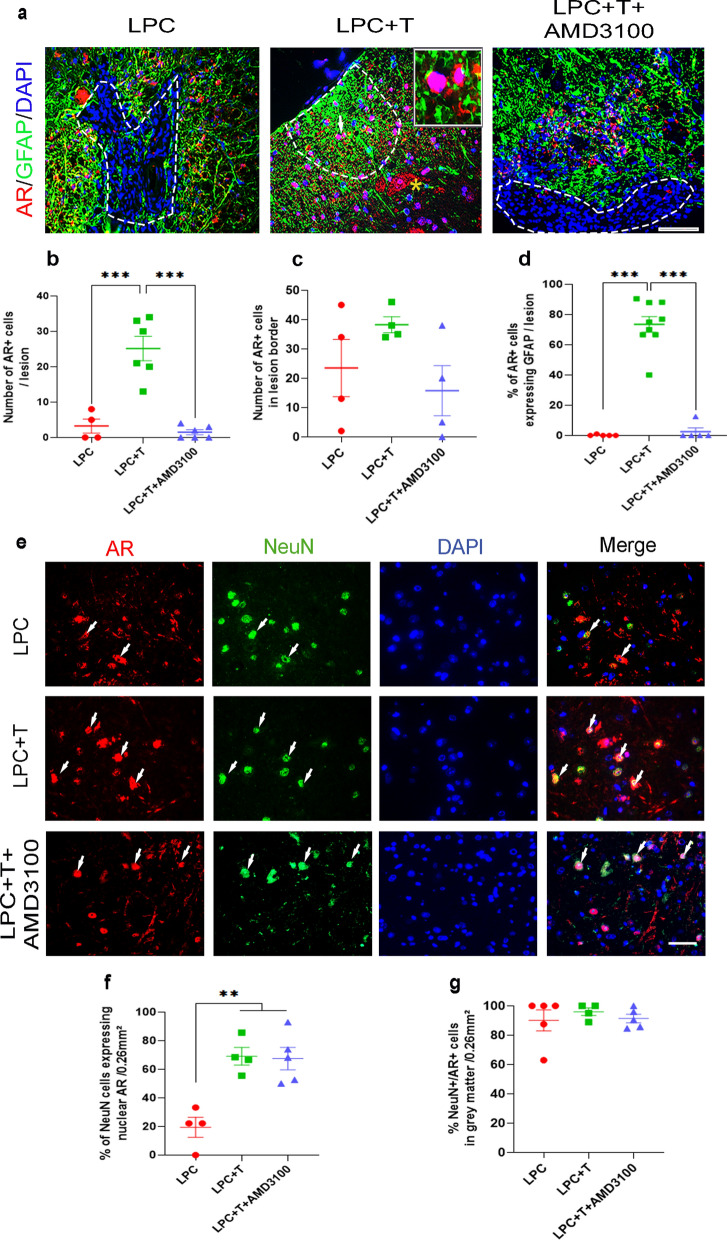
The androgen receptor (AR) is expressed in astrocytes and neurons. **a** Double labeling of AR and GFAP on spinal cord cross sections of castrated males at 30 dpl. Cell nuclei were counterstained in blue with DAPI. Within the LPC lesion, delimited by the dotted line, AR and GFAP staining were very low in males receiving an empty implant (LPC), but AR and GFAP colocalized in males receiving an implant filled with T (LPC + T). Yellow asterisk indicates a motoneuron (**b**–**d**). The enlargement in the LPC + T panel shows AR expression in GFAP^+^ cells. AMD3100 blocked the T-dependent appearance and colocalization of AR and GFAP inside the lesion. AR immunostaining remained present around the lesion after the apparition of AR^+^ astrocytes in the lesion (**c**). **e**–**g** Colocalization of AR and NeuN^+^, marking spinal motoneurons. T treatment was associated with the translocation of neuronal AR into the nucleus and the appearance of nuclear AR immunostaining was not prevented by AMD3100 (**e**, **f**). AR immunostaining in neurons was not affected by treatment with T and AMD3100 (**g**). Data are presented as means ± S.E.M. (one-way ANOVA with Tukey's multiple comparisons tests). Asterisks mark significant differences. ***P < 0.001, **P < 0.01. Scale bars: 20 µm (**a**) and 10 µm (**e**)

A key role of the androgen receptor (AR) in the remyelination signaling of T has been demonstrated by its pharmacological blockade or its genetic removal from the CNS [14, 28]. Here we show that GFAP^+^ astrocytes surrounding the demyelinated LPC lesion and those occurring in the lesion express AR at 30 dpl (Fig. 3a–c). AMD3100, which inhibits the T-dependent presence of activated astrocytes within the demyelinated lesion also prevented the appearance of AR^+^ cells (Fig. 3a, b) and of AR^+^ astrocytes (Fig. 3a, d) inside the lesion. Already at 10 dpl, T increases AR immunostaining within the LPC-lesion (Additional file 1: Fig. S1). The specificity of AR immunostaining was demonstrated by its complete absence in Purkinje cells of cerebellar sections from AR^NesCre^ mice when compared to control wildtypes (Additional file 1: Fig. S2a). This is the first demonstration of AR expression by astrocytes during remyelination, AR having previously mainly been localized to spinal motoneurons [29]. The NeuN^+^ motoneurons in the vicinity of the LPC lesion were indeed immunopositive for AR. In response to T treatment, nuclear translocation of neuronal AR was significantly increased, indicating active T signaling in neurons (Fig. 3e–f). Interestingly, inhibition of CXCR4 signaling by AMD3100 did not affect the T-stimulated nuclear translocation of AR in neurons, although they are well known to express CXCR4 [22] (Fig. 3f). Moreover, the percentage of AR^+^ neurons was not affected by T or AMD3100 treatment (Fig. 3g).

### Inhibition of CXCR4 blocks testosterone-dependent remyelination in both males and females

**Fig. 4:**
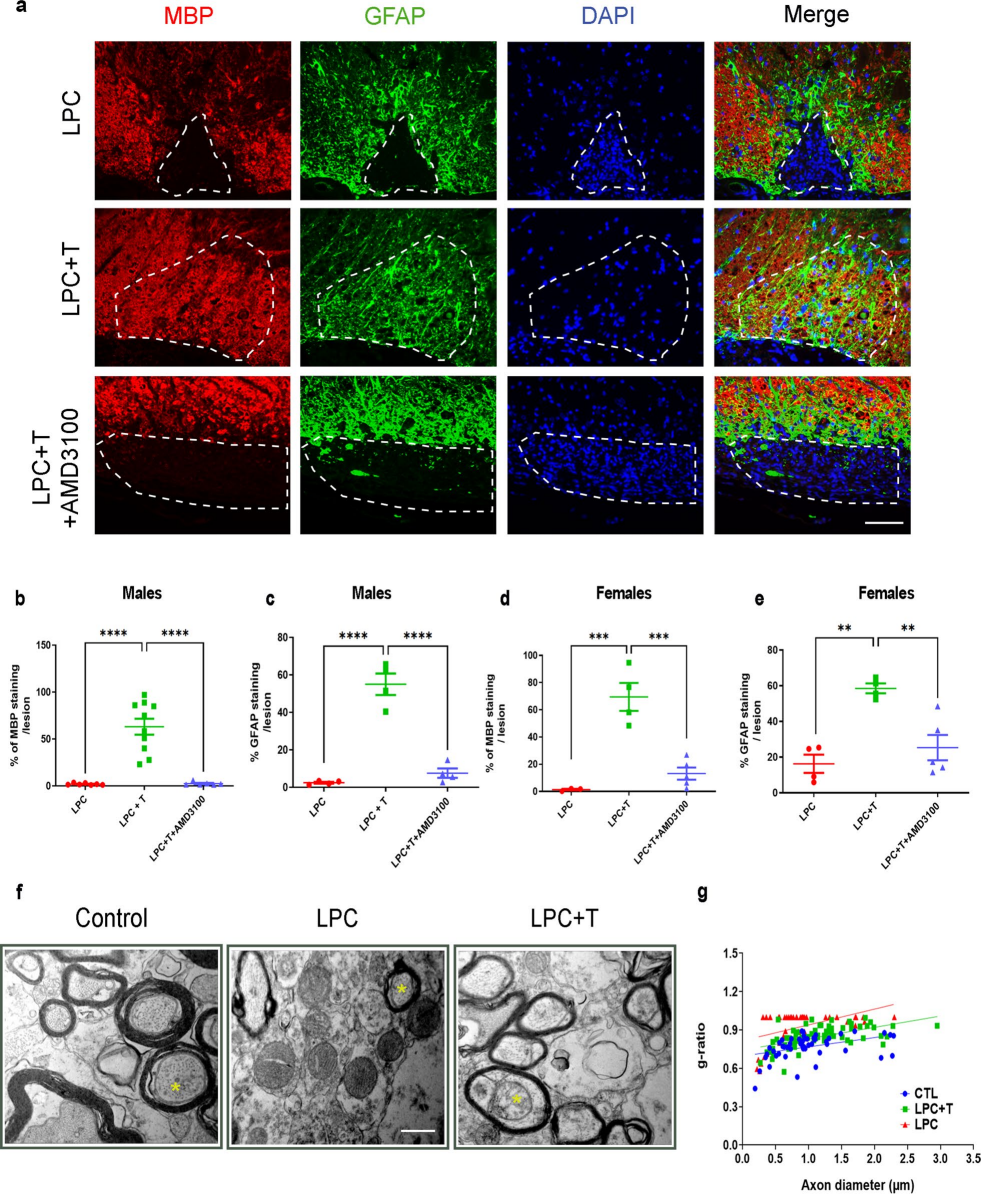
Inhibition of CXCR4 blocks testosterone-dependent remyelination. **a**–**c** Double labeling of MBP and GFAP on spinal cord cross sections of castrated males at 30 dpl. Cell nuclei were counterstained in blue with DAPI. Within the LPC lesion, delimited by the dotted line, both MBP (**a**, **b**) and GFAP (**a**, **c**) staining were nearly absent in males receiving an empty implant (LPC), but MBP and GFAP co-occurred in males receiving an implant filled with T (LPC + T). AMD3100 blocked the T-dependent colocalization of MBP and GFAP inside the lesion (LPC + T + AMD3100). **d** and **f**, T also restored MBP (**d**) and GFAP (**e**) immunostaining within the LPC lesion of ovariectomized females, and its effect was inhibited by AMD3100. Data are presented as means ± S.E.M. (one-way ANOVA with Tukey's multiple comparisons tests). Asterisks mark significant differences. ****P < 0.0001 ***P < 0.001, **P < 0.01. Scale bar: 20 µm. **f** Electron microscopy images of axons and their myelin sheaths within the ventral spinal white matter of gonadally intact males (Control), or within the LPC lesion at 30 dpl of castrated males treated or not with T. Yellow stars indicate myelinated axons. Scale bar: 0.5 µm. **g** Correlations between g-ratios and axon diameters in control males and within the LPC lesion of castrated males treated with T or T + AMD3100

Astrocytes support myelin regeneration by oligodendrocytes [30, 31]. To assess whether the T- and CXCL12/CXCR4-dependent appearance of GFAP^+^ astrocytes within the LPC lesion accompanied CNS remyelination by oligodendrocytes, castrated male mice received an empty or a T-filled s.c. Silastic implant. After 4 weeks, both MBP^+^ myelin and GFAP^+^ astrocytes were fully restored within the lesion area of males treated with T. In contrast, no GFAP^+^ and MBP^+^ immunoreactivities were observed in the absence of T, and the T effect was completely blocked by AMD3100, demonstrating a cooperation between the androgen- and chemokine signaling in CNS remyelination (Fig. 4a–c). Interestingly, T treatment restored and AMD3100 also inhibited MBP^+^ and GFAP^+^ immunoreactivities within the LPC lesion of ovariectomized females (Fig. 4d, e). Although the T implant produced similar tissue T levels in females than in males (13.6 ± 1.0 nM), levels of 5α-DHT and estradiol remained below detection limit.

The T-dependent regeneration of myelin sheaths was examined by electron microscopy. The majority of ventral funiculus axons were surrounded by thin compact myelin after 4 weeks of T treatment, whereas most axons were demyelinated in mice receiving an empty implant or in mice treated with T and AMD3100 (Fig. 4f). Moreover, the mean g-ratio of myelinated axons within the lesion was significantly lower in T-treated than in T + AMD3100 treated mice, reflecting enhanced remyelination (Fig. 4g).

### The cooperation between androgen and CXCR4 signaling is required for the recruitment of oligodendrocytes

**Fig. 5:**
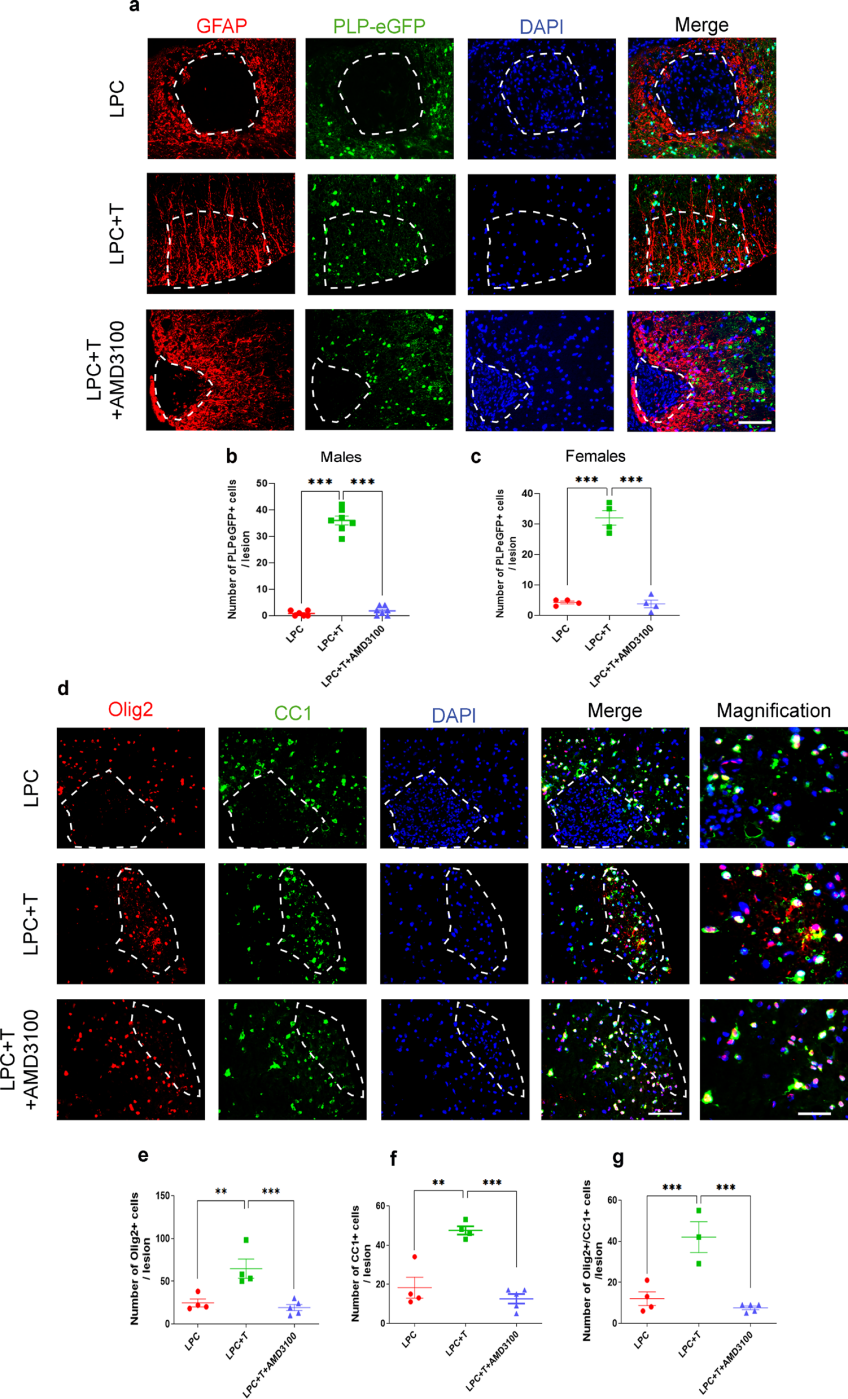
Inhibition of CXCR4 blocks T-dependent recruitment and differentiation of oligodendroglial cells. **a** and **b** Double labeling of GFAP (astrocytes) and eGFP (oligodendroglial cells) on spinal cord cross sections of castrated Plp-eGFP male mice at 30 dpl. Cell nuclei were counterstained in blue with DAPI. Within the LPC lesion, delimited by the dotted line, both GFAP and eGFP staining were absent in males receiving an empty implant (LPC), but in males treated with T, (LPC + T) GFAP^+^ astrocytic processes crossed the lesion, and eGFP^+^ oligodendroglial cells were aligned along them. AMD3100 blocked the T-dependent presence of GFAP immunostaining and eGFP^+^ cells inside the lesion. **c** T also induced the presence of GFAP immunostaining and eGFP^+^ cells within the LPC lesion of ovariectomized females, and its effect was inhibited by AMD3100. **d**–**f** Immunostaining of Olig2 (oligodendroglial cells) and CC1 (mature oligodendrocytes) on spinal cord cross sections of castrated male mice at 30 dpl. Magnification of merged panels show colocalization of Olig2 and CC1. Within the LPC lesion, delimited by the dotted line, both Olig2^+^ and CC1^+^ cells were scarce in males treated with LPC and abundant in males treated with LPC + T. The T-dependent recruitment of Olig2^+^ and CC1^+^ cells within the lesion was blocked by AMD3100. **g** Most of the Olig2^+^ cells were mature oligodendrocytes coexpressing CC1 at 30 dpl. Data are presented as means ± S.E.M. (one-way ANOVA with Tukey's multiple comparisons tests). Asterisks mark significant differences. ***P < 0.001, **P < 0.01. Scale bar: 50 µm in **d** merge and 20 µm in **d** magnification

The recovery of MBP-immunoreactive CNS myelin within a demyelinated lesion requires the recruitment of OPC and their differentiation into myelinating oligodendrocytes. To visualize their migration into the LPC lesion, we used castrated PLP-eGFP transgenic mice, expressing the enhanced green fluorescent protein (eGFP) under the control of the myelin proteolipid protein (PLP) gene promoter in oligodendroglial cells [21]. In castrated male mice, no eGFP fluorescent cells were observed within the LPC lesion at 30 dpl, showing that the disappearance of MBP^+^ myelin was accompanied by the depletion of oligodendrocytes. Treatment of the castrated male mice with a Silastic T implant increased the number of eGFP^+^ oligodendroglial cells within the lesion (Fig. 5a, b). Colabeling with GFAP revealed parallel running astrocytic processes crossing the entire LPC lesion, along which the eGFP^+^ oligodendroglial cells were aligned, suggesting their migration along astrocytic fibers (Fig. 5a). Inhibition of CXCL12/CXCR4 signaling by AMD3100 prevented the T-dependent recruitment of oligodendroglial cells and the formation of astrocytic processes (Fig. 5a, b). Likewise, T increased the number of eGFP^+^ oligodendroglial cells within the LPC lesion in ovariectomized females (Fig. 5c).

Cells present during remyelination also expressed the oligodendrocyte lineage transcription factor 2 (Olig2) (Fig. 5d, e). Double-staining with CC1, a marker of differentiated oligodendrocytes, showed that most Olig2^+^ cells were mature oligodendrocytes (Fig. 5d, f, g). As expected, only very few Olig2^+^ and CC1^+^ oligodendrocytes were present in the lesion of mice treated with T and AMD3100. The density of OPC characterized by the expression of platelet-derived growth factor receptor α (PDGFRα^+^) within the area of remyelination was also significantly increased by T at 30 dpl, and also at an early stage of remyelination (10 dpl), consistent with their T-dependent recruitment (Additional file 1: Fig. S1 and S3a, d). These PDGFRα^+^ OPC also expressed CXCR4 (Additional file 1: Fig. S1 and Fig. S3a, e), as did Olig2^+^ cells (Additional file 1: Fig. S3b, f). Previous studies have indeed shown that CXCL12/CXCR4 signaling plays an important role in the migration, proliferation and differentiation of OPC into mature oligodendrocytes [32–34]. Moreover, mature CC1^+^ oligodendrocytes expressed AR (Additional file 1: Fig. S3c, g).

### Testosterone stimulates the differentiation and migration of oligodendroglial cells in organotypic cultures in a CXCR4-dependent manner

Further evidence in support of a key role for both testosterone (T) and CXCR4 in the differentiation and migration of oligodendroglial cells was obtained in slice cultures of mouse cerebellum and spinal cord. Organotypic cultures faithfully recapitulate the process of myelination with a normal time course, and they provide powerful experimental systems for studying the demyelination and remyelination of axons [27, 35]. We first examined the cooperation between T and CXCR4 in promoting myelin regeneration in cultured spinal cord slices prepared from postnatal day 10 (P10) PLP-eGFP mouse pups (Additional file 1: Fig. S4). The slices were cultured for 7 days in vitro (DIV) to become fully myelinated, and they were then demyelinated by exposing them during 17–18 h to LPC (0.5 mg/ml) as previously described [27]. The culture medium was then changed and slices were treated with vehicle (0.1% ethanol), T (1 µM), AMD3100 (5 µM) or T together with AMD3100 (dosing has been tested). The LPC-treated slices were also compared to controls (CTL), not treated with LPC.

After 5 days, LPC-demyelinated spinal cord slices treated with vehicle were still much depleted in eGFP^+^ oligodendroglial cells, and MBP immunoreactive myelin was sparse when compared to controls (Additional file 1: Fig. S4a–c). Treatment with T restored the density of eGFP^+^ cells and MBP^+^ myelin, becoming comparable to controls. As in vivo, the effects of T were blocked by cotreatment with AMD3100.

**Fig. 6:**
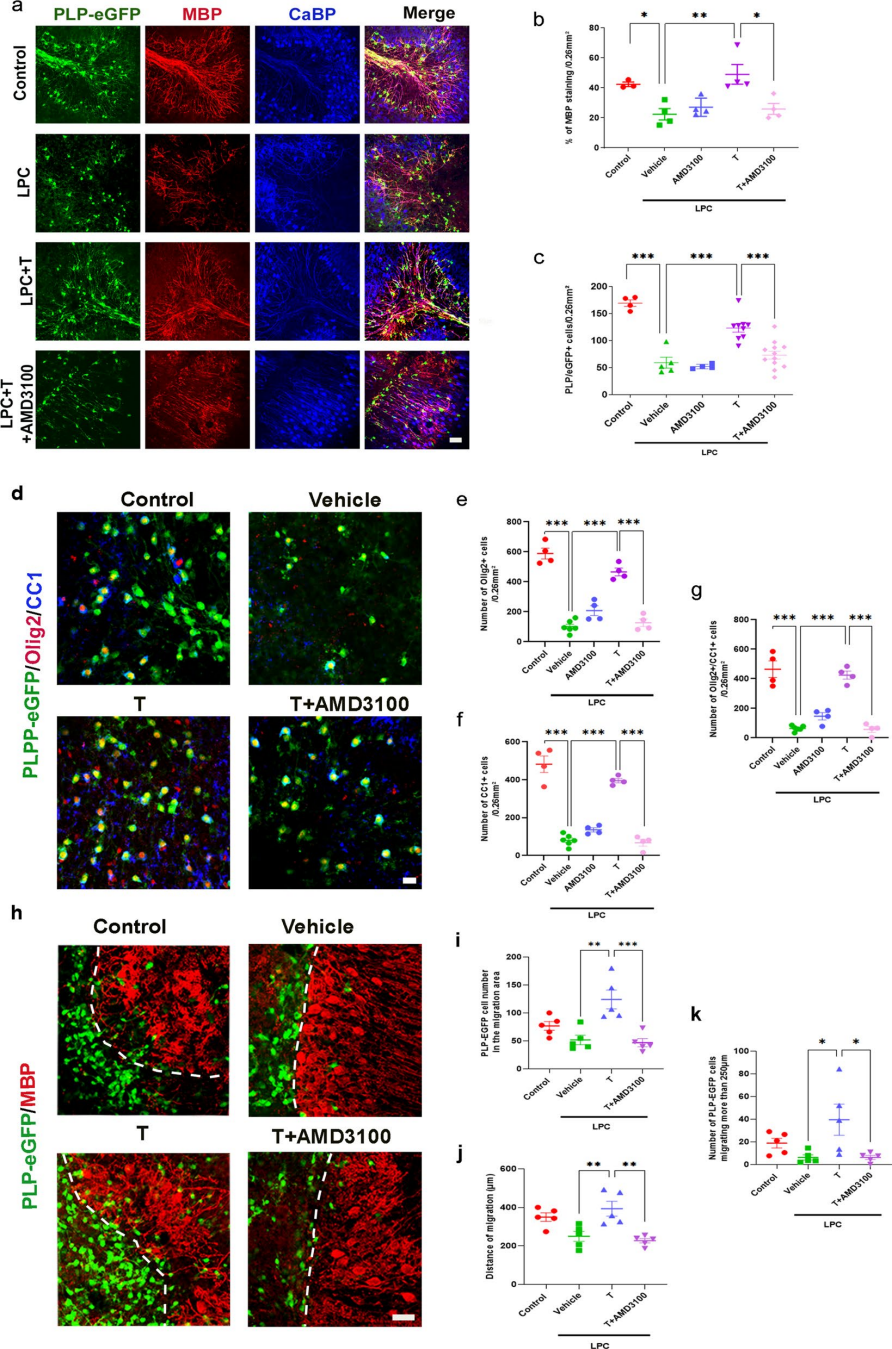
Inhibition of CXCR4 blocks T-dependent remyelination, differentiation of oligodendrocytes and their migration in organotypic cultures of cerebellar slices. **a** Organotypic cultures of sagittal cerebellar slices from postnatal 10 (P10) PLP-eGFP mice. They were triple-stained for eGFP (oligodendroglial cells), MBP (CNS myelin) and calbindin (Purkinje neurons). Control (CTL) slices were only exposed to vehicle (0.1% ethanol). Treated slices were exposed to 0.5 mg/ml LPC for 17–18 h to cause their demyelination. They were then treated with vehicle, T (1 µM) or T + AMD3100 (5 µM) during 5 days. The density of MBP immunostaining and of eGFP ^+^ oligodendroglial cells were quantified. **a**–**c** Slices treated with vehicle or AMD3100 alone remained largely demyelinated (**b**) depleted of eGFP^+^ oligodendroglial cells (**c**). T treatment restored MBP^+^ myelin and replenished eGFP^+^ cells. The effects of T were inhibited by AMD3100. For differentiation and migration of oligodendrocytes, cerebellar slices from postnatal 10 (P10) PLP-eGFP mice were triple stained for eGFP, Olig2 (both markers of oligodendroglial lineage cells) and CC1 (maker of mature oligodendrocytes). Treated slices were exposed to 0.5 mg/ml LPC for 17–18 h to cause their demyelination. They were then treated with vehicle, T (1 µM) or T + AMD3100 (5 µM) during 5 days. **d**–**f** Slices treated with vehicle or AMD3100 alone remained largely depleted of Olig2^+^ cells and CC1^+^ mature oligodendrocytes. T treatment during 5 days replenished Olig2^+^ cells and CC1^+^ cells. **g** Nearly all Olig2^+^ cells coexpressed CC1. The effects of T were inhibited by AMD3100. **h**–**k** Coculture of demyelinated cerebellar slices from P10 C57BL/6 mice and of brain stem slices from P0 PLP-eGFP mice. The dorsal parts of LPC-demyelinated cerebellar slices were apposed with portions of P0 brain stem slices. The dashed line indicates the limit between the two slices. After 10 days of contact without treatment (control) or treatment with vehicle (0.1% ethanol), T (1 µM) or T + AMD3100 (5 µM), the cocultures were immunostained for MBP. T treatment increased the number eGFP^+^ oligodendroglial cells migrating from the brain stem slices into the demyelinated cerebellar slices (**i**), rose their distances of migration (**j**) and significantly increased the number of eGFP^+^ cells migrating a distance greater than 250 µm beyond the border (**k**). These effects of T were inhibited by AMD3100. Data are presented as mean ± S.E.M. (one-way ANOVA with Tukey's multiple comparisons tests). Asterisks mark significant differences. ***P < 0.001, **P < 0.01, *P < 0.05. Scale bar: 20 µm

The effects of T treatment and CXCR4 inhibition by AMD3100 were also examined in LPC-demyelinated slices of mouse cerebellum, because of its precise cellular organization with defined myelinated fiber tracts. Cerebellar slices were prepared from P10 PLP-eGFP mice and treated like the spinal cord slices. The preserved cellular structures in these cultures revealed that eGFP^+^ oligodendroglial cells and MBP^+^ processes were aligned along CaBP^+^ Purkinje cell axons in control and in LPC-demyelinated slices treated during 5 days with T. In contrast, in LPC-demyelinated slices treated with vehicle or concurrently with T and AMD3100, both eGFP^+^ cells and MBP immunoreactivity were scant (Fig. 6a–c).

To assess the cooperation between T and CXCR4 in the replenishment of oligodendroglial cells and their differentiation into mature oligodendrocytes, we double immunostained the cerebellar slices of PLP-eGFP mice with an antibody against the transcription factor Olig2, expressed throughout the oligodendroglial lineage, and an antibody against the CC1 marker of mature oligodendrocytes. After LPC demyelination, T significantly increased the number of mature oligodendrocytes coexpressing eGFP, Olig2 and CC1 to levels observed in control slices (CTL) that were not demyelinated by LPC (Fig. 6d–g). Most Olig2^+^ oligodendroglial cells coexpressed CC1. Thus, T stimulated the replenishment of oligodendroglial cells and their differentiation into mature oligodendrocytes after LPC-induced demyelination. The effects of T required cooperation with CXCR4 signaling, as they were inhibited by AMD3100 (Fig. 6d–g).

The recruitment of new myelinating oligodendrocytes requires not only the proliferation and differentiation of their progenitors, but also their migration into areas of demyelination. To assess whether the cooperation between T and CXCR4 is also involved in oligodendroglial cell migration, P10 cerebellar slices of wildtype mice after 7 DIV were used as controls or demyelinated as described above with LPC. They were then apposed to brain stem slices of newborn (P0) PLP-eGFP mice, which are rich in eGFP^+^ oligodendroglial cells at this developmental stage. This coculture system allowed us a more precise analysis of the migrating eGFP^+^ oligodendroglial cells and their fate. The cocultures of LPC-demyelinated cerebellar slices and P0 brain stem slices were then treated with vehicle, T or T + AMD3100. After 10 days of coculture, the number of eGFP^+^ oligodendroglial cells that had migrated from the brainstem into the cerebellar slices were counted, and the distances travelled from the border of the apposed slices were measured. T treatment increased both the number of migrating oligodendroglial cells and the distances covered by them (Fig. 6h–k). There were 6 times more oligodendroglial cells that migrated a distance greater than 250 µm beyond the border in the presence of T (Fig. 6k). The promigratory effect of T was once again inhibited by AMD3100. Thus, T and CXCR4 increased in a concerted manner the migration, proliferation and differentiation of oligodendroglial cells.

### Astrocytic CXCR4 and AR plays a key role in the testosterone-dependent recruitment of GFAP^+^ astrocytes and the recovery of MBP^+^ myelin

Selective pharmacological blockade of CXCL12/CXCR4 signaling by AMD3100 prevented the T-dependent recruitment of CXCR4^+^ astrocytes inside the demyelinated area and the restoration of MBP^+^ CNS myelin by oligodendrocytes. To assess the role of astrocytic CXCR4 in the recruitment of reactive astrocytes and remyelination, we generated CXCR4^GFAPCre^ mice with selective ablation of CXCR4 in astrocytes using GFAP^Cre^ line 77.6. This line is particularly useful to target astrocytes throughout the healthy and injured brain and spinal cord, with only some off-target Cre recombinase activity observed in stem cells of the subventricular zone [22, 23]. Selective ablation of CXCR4 in astrocytes was confirmed by the loss of CXCR4 immunolabeling in astrocytes of the spinal cord of CXCR4^GFAPCre^ mice (Additional file 1: Fig. S2b, d), whereas NeuN^+^ neurons, PDGFα^+^ OPC and Iba-1^+^ microglial cells continued to express CXCR4 (Additional file 1: Fig. S2c, d).

**Fig. 7:**
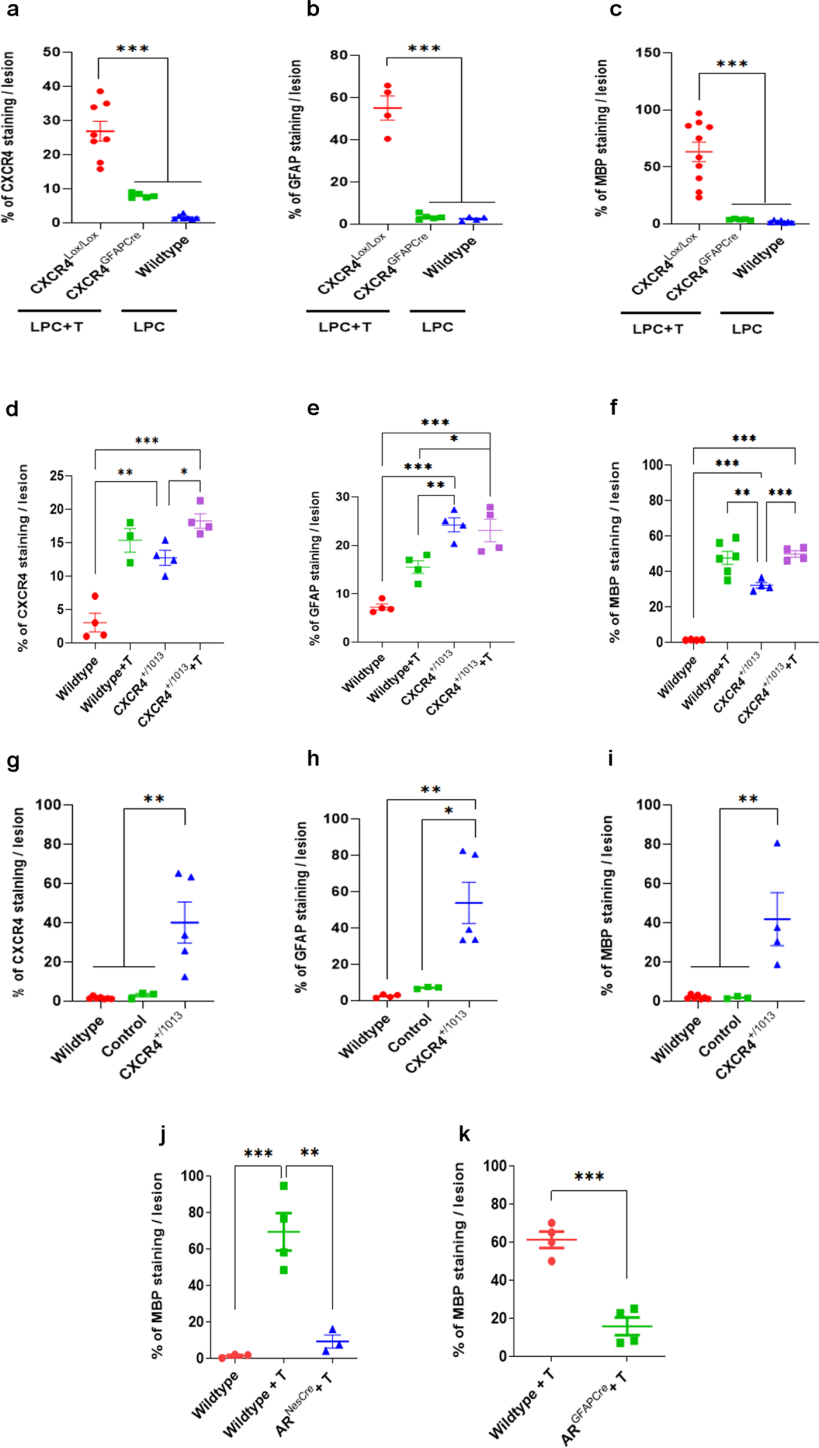
Ablation of CXCR4 and AR in astrocytes and CXCR4 gain of function. **a**–**c** CXCR4 (**a**), GFAP (**b**) and MBP (**c**) immunostaining were quantified within the LPC lesion at 30 dpl of castrated male CXCR4^GFAPCre^ (inactivation of CXCR4 in astrocytes), CXCR4^Lox/Lox^ (exon 2 LoxP-flanked) and unfloxed wildtype mice. CXCR4^GFAPCre^ and CXCR4^Lox/Lox^ mice were treated during 4 weeks with a T-filled implant, whereas the wildtypes received an empty implant. **d**–**f**, CXCR4 (**d**), GFAP (**e**) and MBP (**f**) immunostaining were quantified within the LPC lesion at 15 dpl of castrated male CXCR4^+/1013^ mice (heterozygous CXCR4 gain of function mutation) and wildtype mice receiving an empty or T filled implant. **g**–**i** CXCR4 (**g**), GFAP (**h**) and MBP (**i**) immunostaining were quantified within the LPC lesion at 30 dpl of castrated male wildtype mice, CXCR4^+/1013^ mice and the corresponding littermate control mice. **j**, **k**, A role for astrocytic AR in the remyelinating effects of testosterone. In castrated AR^NesCre^ mice (inactivation of AR within the CNS), T treatment failed to stimulate remyelination within the LPC lesion (**j**). In transgenic AR^GFAPCre^ mice (inactivation of AR in astrocytes), the remyelinating action of T was also significantly reduced (**k**). Data are presented as means ± S.E.M. (one-way ANOVA with Tukey's multiple comparisons tests). Asterisks mark significant differences. ***P < 0.001, **P < 0.01, *P < 0.05

Adult male CXCR4^GFAPCre^, CXCR4^Lox/Lox^ and unfloxed wildtype mice were then castrated and received the same procedure of LPC demyelination. CXCR4^GFAPCre^ and CXCR4^Lox/Lox^ mice were treated for 4 weeks with a T-filled implant, whereas the wildtypes received an empty implant. In the lesion, CXCR4 immunostaining was markedly decreased in the T-treated CXCR4^GFAPCre^ mice when compared to the T-treated CXCR4^Lox/Lox^ controls, which were comparable to the very low levels of wildtypes receiving an empty implant (Fig. 7a). In T-treated CXCR4^Lox/Lox^ mice, elevated CXCR4 expression within the lesion was associated with strong GFAP and MBP immunostaining, whereas astrocyte ablation of CXCR4 resulted in very low GFAP and MBP levels (Fig. 7b, c). These results demonstrate a key role of astrocytic CXCR4 in the appearance of astrocytes in the demyelinated area and the recovery of MBP^+^ myelin.

Complementary to this experiment, the consequences of CXCR4 gain of function were examined in CXCR4^+/1013^ mice with the heterozygous CXCR4 mutation characteristic of the Warts, Hypogammaglobulinemia, Infections, and Myelokathexis (WHIM) syndrome. This mutation results in enhanced and prolonged CXCR4 responses to CXCL12 [24]. Adult male CXCR4^+/1013^ mice, their littermate controls and wildtype C57BL6/6J mice were castrated and after LPC lesion, they were treated for 15 days with an empty or T-filled implant to assess the early effects of CXCR4 gain of function in the absence or presence of T.

In the absence of T, CXCR4 immunostaining within the LPC lesion of CXCR4^+/1013^ mice was comparable to T-induced levels in wildtypes. Treatment of CXCR4^+/1013^ mice with T further increased CXCR4 immunolabeling (Fig. 7d). Of note, CXCR4 gain of function was sufficient, even in the absence of T, for astrocyte appearance in the lesion and some MBP^+^ myelin increase (Fig. 7e–f). In contrast, the appearance of GFAP^+^ astrocytes within the lesion was dependent on T in wildtype mice (Fig. 7e). However, although GFAP^+^ astrocytes occupy the lesion in CXCR4^+/1013^ mice in the absence of T, treatment with T significantly increased MBP^+^ myelin in both wildtype and CXCR4^+/1013^ mice (Fig. 7f). Thus, CXCR4 gain of function is beneficial to but not sufficient for optimal remyelination at 15 dpl, requiring cooperativity between CXCR4 and androgen signaling. At 30 dpl, CXCR4 gain of function allowed a significant rescue of myelin loss, even in the absence of T treatment. This was shown by treating wildtype, control and CXCR4^+/1013^ mice with an empty implant during 4 weeks after LPC (Fig. 7g–i). However, MBP immunostaining in CXCR4^+/1013^ mice remained below MBP levels observed when wildtype mice were treated by T (compare Fig. 7i to Figs. 1a and 4b).

To assess whether AR expression in astrocytes is also required for the remyelinating effect of testosterone, we first verified that in castrated AR^NesCre^ mice with CNS-selective AR ablation, T treatment failed to stimulate the recovery of MBP^+^ myelin within the LPC lesion (Fig. 7j). In AR^GFAPCre^ mice with selective ablation of AR in astrocytes, the remyelinating action of T was also significantly reduced, demonstrating a key role of astrocytic AR in the recovery of MBP^+^ myelin (Fig. 7k). Thus, expression of both CXCR4 and AR in astrocytes is required for efficient myelin regeneration.

### Schwann cells replenish the demyelinated lesion and remyelinate axons in the absence of AR and CXCR4 signaling

**Fig. 8:**
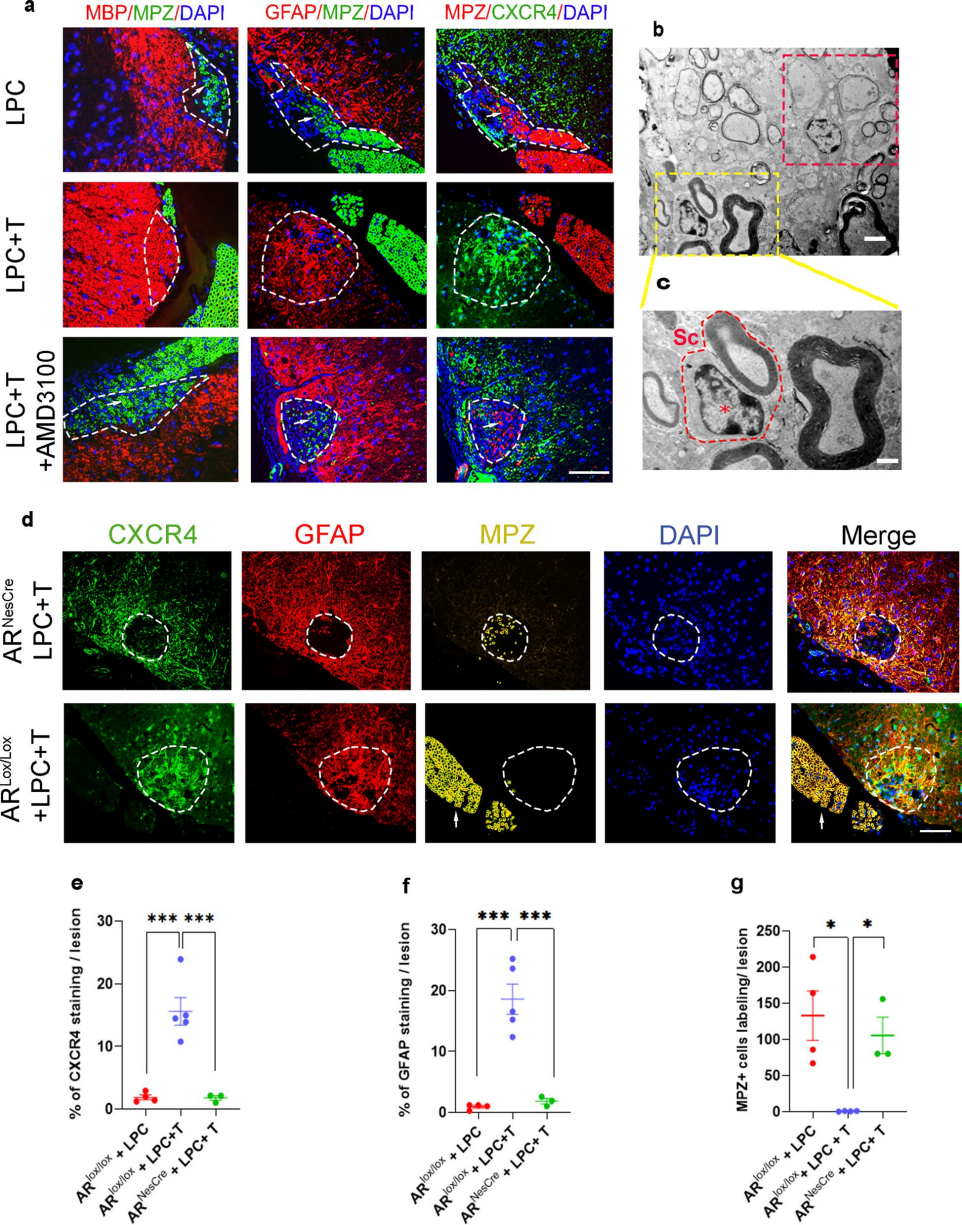
Schwann cell remyelination takes place in the absence of CXCR4 or AR signaling. **a** MPZ/MBP, MPZ/GFAP and MPZ/CXCR4 double-labeling on spinal cord cross sections of wild-type castrated AR^Lox/Lox^ male mice at 30 dpl. Cell nuclei were counterstained in blue with DAPI. The LPC lesion is delimited by a dotted line. MPZ^+^ Schwann cells, most likely originating from the ventral spinal roots (indicated by arrows) invaded the lesion only in the absence of MBP, GFAP or CXCR4 immunostaining, which means in the absence of T (empty implant) or CXCR4 signaling (AMD3100). Electron microscopy image (**b**) and its enlargement (**c**) showing that in the absence of T, axons are remyelinated by Schwann cells (Sc), characterized by a large nucleus close to the myelin sheaths (indicated by a star). Red square indicates an oligodendrocyte in the lesion in contact with two axons, one axon presents a thin myelin sheaths and a second axon seems to be unmyelinated. **d**–**g** CXCR4, GFAP, and MPZ immunostaining of spinal cord cross sections of castrated AR^Lox/Lox^ and AR^NesCre^ male mice at 30 dpl. Transgenic AR^NesCre^ mice with CNS-selective ablation of AR and control AR^Lox/Lox^ mice were used. The LPC lesion is delimited by a dotted line. MPZ^+^ Schwann cells, most likely originating from the ventral spinal roots (indicated by arrows), invaded the lesion only in the absence of CXCR4 and GFAP immunostaining (AR^Lox/Lox^ mice receiving an empty implant or AR^NesCre^ mice receiving a T implant). Scale bar: 20 µm in (**a**, **d**) and 1 µm in (**b**, **c**). **e**–**g** Data are presented as means ± S.E.M. (one-way ANOVA with Tukey's multiple comparisons tests). Asterisks mark significant differences. ***P < 0.001, *P < 0.05

In the absence of astrocytes, axons have been shown to undergo remyelination by Schwann cells [36, 37]^.^ Also, paucity of astrocytes resulting from the absence of T or a functional AR, led to extensive Schwann cell remyelination [14]. At 30 dpl, when GFAP^+^ astrocytes and MBP^+^ CNS myelin remained sparse within the demyelinating lesion of castrated males, axons were remyelinated by MPZ^+^ peripheral nervous system (PNS) myelin (Fig. 8A). Electron microscopy revealed that axons were remyelinated by Schwann cells, which are normally exclusively present in the PNS. They were identified by the presence of a large nucleus adjacent to the myelin (Fig. 8b, c). As expected, GFAP^+^ and CXCR4^+^ astrocytes were recruited, and MBP^+^ CNS myelin was restored at the exclusion of MPZ^+^ PNS myelin when castrated males were treated with T (Fig. 8a). AMD3100 inhibited the T-dependent recovery of activated astrocytes and MBP^+^ myelin within the demyelinated lesion, giving way to MPZ^+^ Schwann cell remyelination. Immunohistochemistry images suggested that in the absence of T or after inhibiting CXCR4 with AMD3100, the lack of astrocytes allowed MPZ^+^ Schwann cells to migrate into the lesion from nearby ventral spinal nerve roots (Fig. 8a, d).

The T-dependent appearance of GFAP and CXCR4 expressing astrocytes in the demyelinated lesion and the exclusion of spinal nerve root Schwann cells involved a functional AR, as Schwann cell instead of oligodendrocyte remyelination took place in AR^NesCre^ mice with CNS-selective ablation of AR (Fig. 8d–g). Dual immunolabeling of MPZ and the large axon marker NF200 showed that only part of the axons was remyelinated by Schwann cells (about 50%). As in males, Schwann cells invaded the LPC-induced lesion in ovariectomized females in the absence of T or after blocking CXCR4 with AMD3100, and Schwann cell remyelination was inhibited by T (Additional file 1: Fig. S5b, c).

### Astrocytes expressing CXCR4 and AR exclude Schwann cells in the area surrounding MS lesions

To further investigate the significance of our experimental findings for demyelinating diseases, we performed different combinations of CXCR4, AR, MBP, GFAP and MPZ immunolabeling on autopsied spinal cord sections from 5 men and 5 women with secondary progressive (SP) or primary progressive (PP) multiple sclerosis (MS). Provided by the Netherland Brain Bank (NBB), the age of the donors ranged from 53 to 69 years, the postmortem delay did not exceed 10 h, and importantly, the narrow postmortem cerebrospinal fluid pH range (6.14–6.88) ensured good histological and molecular tissue preservation [38] (Additional file 1: Table S2).

**Fig. 9:**
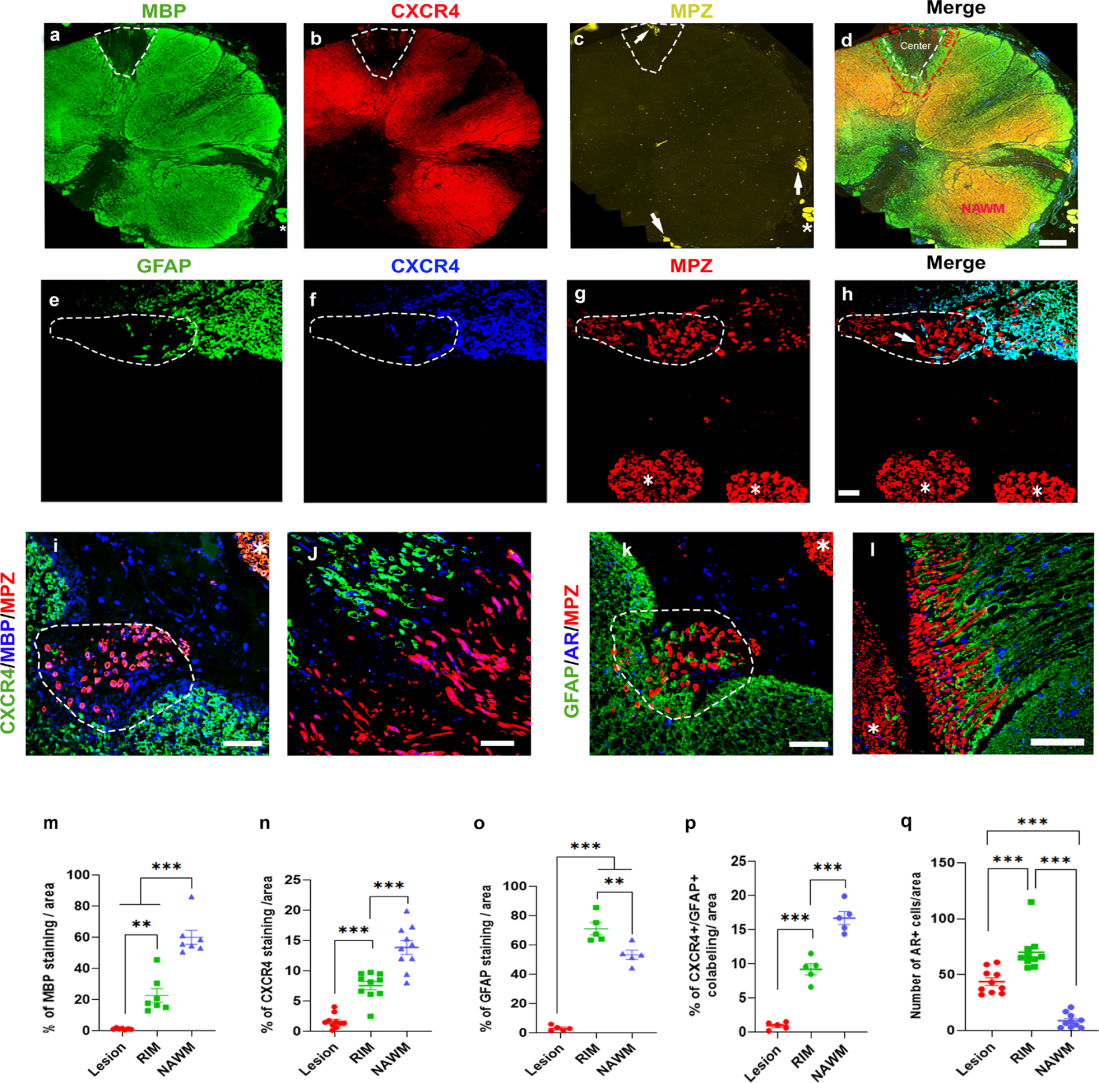
Astrocytes, AR and CXCR4 are highly expressed at the border of human white matter MS lesions. **a**–**d** Coronal section of the thoracic spinal cord from a female MS patient with 3 mixed active/inactive (chronic active) lesions were immunostained for MBP, CXCR4 and MPZ. The center and RIM of a lesion are delimited by dotted lines. NAWM = Normal Appearing White Matter. Scale bar: 1 mm. **e**–**h** Enlarged image of a lesion RIM immunostained for GFAP, CXCR4 and MPZ. GFAP and CXCR4 colocalized, indicating CXCR4 expressing astrocytes, and their immunostaining never overlapped with the Schwann cell marker MPZ (arrows). The MPZ^+^ Schwann cells most likely originated from nearby spinal nerve roots (indicated by stars). Scale bar: 30 µm. **i** and **j** Enlarged view of the colocalization of CXCR4 and MBP at the RIM, which was excluded of MPZ^+^ immunostaining. Scale bar: 50 µm. **k** and **l** GFAP and AR immunostaining colocalized and were exclusive to MPZ^+^ Schwann cells, most likely originating from nearby spinal nerve roots (star). Scale bar: 50 µm. **m**–**p** Quantification of MBP (**m**), CXCR4 (**n**), GFAP (**o**) and CXCR4/GFAP immunostaining (**p**) and counting of AR^+^ cells (**q**) within the lesion center, lesion RIM and NAWM of the MS lesions identified on spinal cord sections from the 10 MS patients (5 men and 5 women). Lesion is delimited by the dotted line. Data are presented as means ± S.E.M. (one-way ANOVA with Tukey's multiple comparisons tests). Asterisks mark significant differences. ***P < 0.001, **P < 0.01

Immunohistochemistry analysis was performed in the center and border (RIM) of active and mixed active/inactive (chronic active) lesions as well as in normal-appearing white matter (NAWM) (Fig. 9a–q). MBP immunolabeling was very low within the center, elevated in the RIM and even higher in NAWM (Fig. 9m). CXCR4 staining showed a similar pattern, reflecting the overlap of MBP and CXCR4 immunostaining in RIM and NAWM (Fig. 9a–d, i, j). Remarkably, CXCR4 labeling was highest in the 2 identified remyelinated shadow plaques (Additional file 1: Fig. S6a).

In line with our experimental observations in mouse brain, the presence of MBP^+^ myelin concurred with GFAP^+^/CXCR4^+^ astrocytes in perilesional areas and NAWM, but was nearly absent in the myelin-poor MS lesion centers (Fig. 9a–j). CXCR4 and GFAP indeed colocalized in the RIM of MS lesions, indicating CXCR4 expression in activated astrocytes surrounding areas of demyelination (Fig. 9p). GFAP immunostaining was even slightly but significantly higher in RIM than in NAWM (Fig. 9o). Moreover, RIM GFAP^+^ astrocytes also expressed AR, consistent with cooperative androgen and chemokine signaling in the perilesional area, also containing MBP^+^ myelin (Fig. 9k, l). Of note, the number of AR^+^ cells was higher in lesion RIM when compared to the lesion center and in particular to NAWM, suggesting an upregulation of the receptor in response to demyelination (Fig. 9p). For these analyses, data of men and women were pooled as there were no differences between sexes for CXCR4 immunolabeling and the density of AR^+^ cells in the center or at the borders of MS lesions (Additional file 1: Fig. S6b–e).

In MBP-sparse regions of MS lesions, infiltration of MPZ^+^ Schwann cells was observed (Fig. 9g). GFAP/CXCR4/MPZ labeling in MS perilesional areas showed that GFAP^+^/CXCR4^+^ immunostaining was exclusive to MPZ^+^ immunostaining (Fig. 9a–h), as were CXCR4^+^/MBP^+^ (Fig. 9i, j) and GFAP^+^/AR^+^ immunostaining (Fig. 9k, l). Thus, as in demyelinating lesions of the mouse spinal cord, the presence of CXCR4 and AR expressing astrocytes appear to oppose the arrival of MPZ^+^ Schwann cells at the border of MS lesions, most likely originating from nearby spinal roots (stars). The number of MPZ^+^ cells entering MS lesions did not differ between males and females (Additional file 1: Fig. S6f).

## Discussion

Sex differences in the incidence and progression of MS provided the rationale for earlier studies testing the effects of T therapy in EAE [39]. Their beneficial outcomes, ascribed to the immunomodulatory and neuroprotective effects of T [40], prompted a pilot trial showing protective effects of transdermal testosterone in men with MS [41, 42]. The growing interest in regenerative therapies for restoring lost myelin [43] then encouraged us to study the role of T in myelin repair. We demonstrated strong remyelinating actions of T involving AR in models of toxin-induced demyelination [11–14]. We also showed that T stimulates developmental myelination [28]. Moreover, we recently showed that even in females AR signaling promotes myelin repair by regulating neuroinflammatory responses [12].

Here, we explored concerted actions of T and CXCR4 signaling in myelin regeneration after LPC infusion into the ventral white matter of the mouse spinal cord. When well-dosed, this phospholipid selectively destroys myelin and kills oligodendrocytes and astrocytes within a focal area [44, 45]. Gene microarray analysis within microdissected LPC lesions, at the beginning of the remyelination process, identified CXCR4 as a major T-upregulated gene. Androgens have been shown to regulate CXCR4 expression in prostate cancer cells, either directly by putative androgen-responsive elements (ARE) in the CXCR4 gene promoter [46], or indirectly by androgen-sensitive transcription factors [47]. Interestingly, ARE have also been identified in the CXCL12 promoter [48], suggesting that T may be an upstream regulator of the CXCL12/CXCR4 axis. In favor of such an interpretation was the observation that in CXCR4^+/1013^ mice with a heterozygous CXCR4 gain of function mutation characteristic of WHIM syndrome, remyelination was observed even in the absence of testicular T.

The recovery of MBP-immunoreactive CNS myelin within the LPC lesion was paralleled by the appearance of astrocytes. Moreover, the induction of CXCR4 and CXCL12 by T within the demyelinating lesion matched the accumulation of astrocytes, expressing CXCR4 and CXCL12 together with AR, indicating autocrine/paracrine chemokine and androgen signaling mechanisms.

**Fig. 10:**
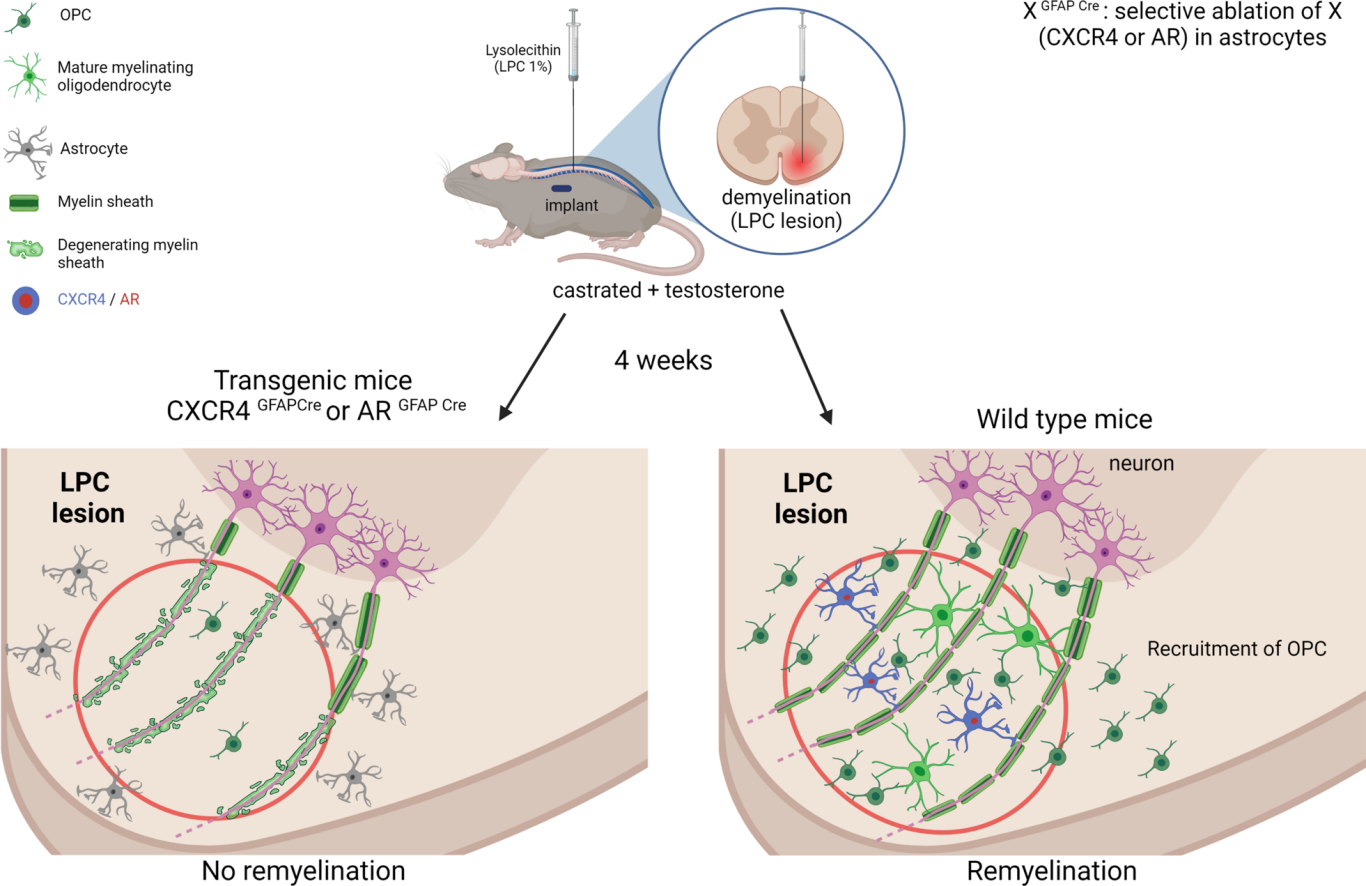
Schematic illustration of potential role of astrocytic CXCR4 and AR in the remyelinating effect of testosterone. After focal demyelination produced in the ventral mouse spinal cord by the infusion of lysolecithin, the testosterone-dependent remyelination of axons by oligodendrocytes was accompanied by an increase in astrocytes expressing CXCR4 and the androgen receptor within the demyelinated area. Conditional ablation of CXCR4 or AR in astrocytes blocked remyelination by oligodendrocytes

Particularly striking was the complete interdependence of androgen and chemokine signaling in the appearance of astrocytes within the LPC lesion, which accompanied oligodendrocyte replenishment and the formation of new myelin. Depriving mice of their endogenous T by castration, a non-functional AR in all neural cells or only in astrocytes, pharmacological inhibition of the CXCL12/CXCR4 chemokine axis or genetic conditional ablation of CXCR4 within astrocytes indeed all resulted in the complete absence of astrocytes and new myelin formation by oligodendrocytes after LPC. Importantly, in CXCR4^+/1013^ mice, both astrocytes and CNS myelin recovered after castration. Our work thus identifies astrocytes as one of the main cellular mediators of the interplay between T and CXCL12/CXCR4, necessary for myelin repair (Fig. 10).

Where do the astrocytes that repopulate the demyelinating LPC lesion come from? After castration or blocking CXCL12/CXCR4 signaling by AMD3100, astrocytes expressing GFAP and CXCR4 accumulated at the lesion border. In contrast, in the presence of T and CXCR4 signaling, their density decreased at the borders and increased within the lesions. These results could be interpreted as a T- and CXCR4-dependent migration of astrocytes into the demyelinated lesion. However, although LPC has been reported to kill astrocytes [44, 45] and GFAP immunoreactivity disappeared after LPC infusion, spared astrocytes may have contributed to the restoration of astrocytes within the lesion [49, 50]. Whatever the origin of the astrocytes, their processes may guide the migration of OPC. In the presence of T and CXCL12/CXCR4 signaling, astrocytic processes were indeed observed to cross the LPC lesion and along them eGFP^+^ oligodendroglial cells were aligned.

Another glial cell type involved in myelin repair, microglia, is also influenced by the presence of T in the LPC-lesion microenvironment. In the absence of T, microglia followed the behavior of astrocytes, localized more around the lesion, whereas in its presence, microglia was increased within the lesion. It is possible that the maintenance of a glial scar around the lesion in the absence of T disturbs the migration of microglial cells. A reduced recruitment of microglial cells in the absence of T, already observed here at 10 dpl, may hinder the phagocytosis of myelin and cell debris and thus contribute to impaired remyelination. Microglial cells also play an important role in astrocyte proliferation, oligodendrocyte survival and in reducing proinflammatory factors. Importantly, astrocytes, microglial cells, OPC as well as AR and CXCR4 co-expression were already significantly increased within the LPC lesion as early as 10 dpl.

AR are expressed in spinal cord neurons, and neuroprotective effects of T have been documented in different CNS lesion systems [51] and also in men with multiple sclerosis [42]. It is therefore conceivable that T may exert protective effects on neurons during the demyelination process shortly after LPC infusion. However, neither a substantial loss of axons after LPC-induced demyelination, nor a marked increase in axonal density by T treatment have been observed under the here used experimental conditions [14]. On the other hand, trophic effects of T on neurons may have a beneficial influence on the formation of new myelin sheaths, as there are close interactions between axons and oligodendrocytes and as neurons influence myelinogenesis [52]. Here, we demonstrate that the cooperation between AR and CXCR4 signaling in astrocytes is a prerequisite for the formation of new myelin. This essential new mechanism does not rule out additional myelin-potentiating effects T and CXCL12 on neurons, oligodendrocytes and microglial cells.

CXCL12 produced by astrocytes has been proposed to promote the differentiation of CXCR4 expressing OPC into oligodendrocytes, and our results confirm that OPC express CXCR4 [15, 16, 53]. In addition, OPC co-express CXCL12 and CXCR4 [54]. However, autocrine CXCL12/CXCR4 signaling in OPC or paracrine CXCL12/CXCR4 signaling between OPC and neurons are not sufficient for the generation of myelin forming oligodendrocytes as astrocyte-specific silencing of CXCR4 prevented remyelination by oligodendrocytes.

Organotypic cultures of LPC-demyelinated spinal cord and cerebellar slices prepared from mice expressing the eGFP reporter in oligodendroglial cells allowed us to further characterize the reciprocal interactions between T and CXCR4 during major stages of the remyelination process. Addition of T to the culture medium stimulated the replenishment of the demyelinated slices with oligodendrocytes and the recovery of MBP^+^ myelin, and these effects were blocked by AMD3100. T also enhanced in a CXCR4-dependent manner the migration of oligodendroglial cells into demyelinated cerebellar slices.

Whereas oligodendrocytes remyelinated axons in the presence of activated astrocytes in a T- and CXCR4-dependent manner, axons were remyelinated in their absence by Schwann cells. In the CNS, axons can indeed be remyelinated by Schwann cells in the absence of astrocytes [14, 36, 55]. Astrocyte activation also determines the balance of oligodendrocyte versus Schwann cell remyelination [56]. In the spinal cord, Schwann cells derived from spinal nerves can remyelinate axons after the disruption of astrocyte limitans [57].

Immunohistochemical analysis of autopsied spinal cord sections from MS patients provided support for the physiopathological relevance of our experimental findings. No significant differences were found between sexes, and for this reason, results of male and female MS lesions have been combined. Because of the age (53–69 years) and disease status of the MS patients, steroid-depended sex differences may indeed be attenuated. Important observations were the correspondence between CXCR4 and MBP expression in NAWM and at the RIM of active and mixed active/inactive lesions, and the colocalization of CXCR4 with GFAP. Perilesional astrocytes also expressed AR, in accordance with an interdependent astrocytic androgen and chemokine signaling. Strong AR immunoreactivity colocalizing with GFAP^+^ astrocytes has previously been observed in the RIM of MS lesions [58]. Importantly, as in the LPC demyelinated lesions, CXCR4^+^ and AR^+^ astrocytes were exclusive to MPZ^+^ Schwann cells around MS lesions.

The regulation of CXCR4 by T and their cooperative signaling guide to the importance of normalizing low T levels for efficient myelin regeneration therapy. Restoring T levels to age-adjusted physiological levels is indeed associated with multiple health benefits in men [59, 60]. Depending on the patient cohort and clinical progression of MS, the prevalence of serum T levels below the lower limit of normal (< 10 nmol/L) lies between 30 and 80% [5, 6]. Interestingly, T has also beneficial immunological effects relevant for MS [61], and CXCL12/CXCR4 signaling is protective in EAE by preventing lymphocytes from invading the CNS and by restraining autoimmune inflammatory processes [62, 63].

Restoring normal T levels may thus become part of personalized remyelinating treatments and precision medicine in MS.

### Supplementary Information

**Additional file 1.** Table S1-S3 and Fig. S1-S6. Table S1. Representative differentially expressed genes in LPC+T- versus LPC-lesioned mice spinal cords; Fig S1 : Quantification of oligodendrocytes, microglia an androgen receptor at early time points after LPC injection. Table S2. Clinicopathologic Data on Multiple Sclerosis Patients and Controls. Fig S2: Validation of AR antibody specificity and astrocyte-specific genetic inactivation of CXCR4. Table S3. Antibodies used for immunohistochemistry. Fig S3: Oligodendroglial cells express CXCR4 and AR. Fig S4: Inhibition of CXCR4 blocks T-dependent remyelination in organotypic spinal cord cultures. Fig S5: Inhibition of CXCR4 promotes remyelination of the central nervous system by Schwann cells. Fig S6: Comparison of CXCR4, AR and MPZ immunostaining between men and women with MS.

## Abbreviations

GFAP: Glial fibrillary acidic protein
NF200: Neurofilament 200
OPC: Oligodendrocyte progenitor cells
MS: Multiple sclerosis
T: Testosterone
EAE: Experimental autoimmune encephalomyelitis
LPC: Lysolecithin
AR: Androgen receptor
MBP: Myelin basic protein
MPZ: Myelin protein zero
PLP: Myelin proteolipid protein
eGFP: Enhanced green fluorescent protein
PDGFRα^+^: Platelet-derived growth factor receptor α
Olig2: Oligodendrocyte lineage transcription factor 2
CC1: Mature oligodendrocyte
DAPI: 4′,6-Diamidino-2-phénylindole
PBS: Phosphate-buffered saline
