# Correction to: Interplay between androgen and CXCR4 chemokine signaling in myelin repair

**DOI:** 10.1186/s40478-024-01774-3

**Published:** 2024-07-15

**Authors:** Narimene Asbelaoui, Charly Abi–Ghanem, Geraldine Schlecht–Louf, Hania Oukil, Cindy Degerny, The Netherlands Brain Bank, Michael Schumacher, Abdel Mouman Ghoumarir

**Affiliations:** 1 UMR1195, “Diseases and Hormones of the Nervous System”, Inserm and University Paris-Saclay, 80, Rue du General Leclerc, Kremlin–Bicetre,, 94276 Orsay, France; 2 INSERM UMR 996, Inserm, Inflammation, Microbiome and Immunosurveillance, Faculte de Pharmacie, Universite Paris-Saclay, Orsay, France; 3 Netherlands Institute for Neuroscience, Amsterdam, The Netherlands; 4 Present Address: Department of Neuroscience and Experimental Therapeutics, Albany Medical College, Albany, NY USA

**Correction to**: *Acta Neuropathol Commun ***12**, 18 (2024). 10.1186/s40478-024-01730-1.

Following publication of the original article [1], the authors reported that Cindy Degerny was omitted from the author group. Cindy Degerny has been added to the author group.

The updated Author contributions section is given hereafter:

**Author contributions**

Conceptualization: AMG; Methodology: AMG, NA; Investigation: AMG, NA, CAG, GSL, HO; Visualization: AMG, MS; Supervision: AMG, MS; Writing?original draft: NA, AMG; Writing?review & editing: AMG, MS; NBB provided the human MS samples. NBB is represented by Pr. Inge Huitinga. CD has contributed to the differential transcriptomic data presented in Additional file 1: Table S1. All authors read and approved the final manuscript.

The authorlist and Author contributions section has been updated and the original article [1] has been corrected.
