# Supplementary material for: Interplay between androgen and CXCR4 chemokine signaling in myelin repair
Source: Acta Neuropathol Commun. 2024 Jan 30;12:18. doi: 10.1186/s40478-024-01730-1 (PMC10826258; doi:10.1186/s40478-024-01730-1)
Supplement: Supplementary file 1 — Additional file 1. Table S1-S3 and Fig. S1-S6. Table S1. Representative differentially expressed genes in LPC+T- versus LPC-lesioned mice spinal cords; Fig S1 : Quantification of oligodendrocytes, microglia an androgen receptor at early time points after LPC injection. Table S2. Clinicopathologic Data on Multiple Sclerosis Patients and Controls. Fig S2: Validation of AR antibody specificity and astrocyte-specific genetic inactivation of CXCR4. Table S3. Antibodies used for immunohistochemistry. Fig S3: Oligodendroglial cells express CXCR4 and AR. Fig S4: Inhibition of CXCR4 blocks T-dependent remyelination in organotypic spinal cord cultures. Fig S5: Inhibition of CXCR4 promotes remyelination of the central nervous system by Schwann cells. Fig S6: Comparison of CXCR4, AR and MPZ immunostaining between men and women with MS. [file 40478_2024_1730_MOESM1_ESM.docx]

**Supplemental data, figures and tables:**

**
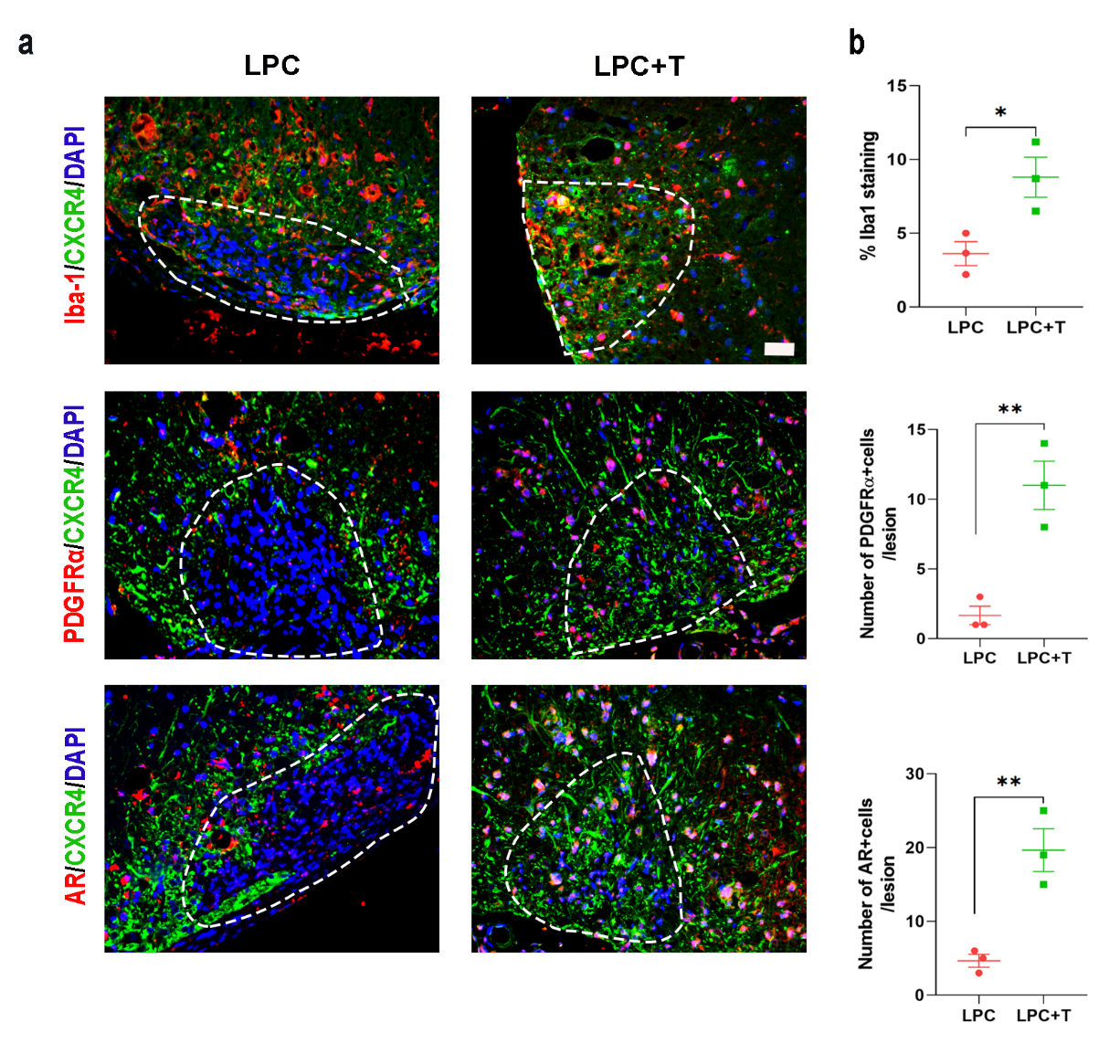
Supplemental Fig. 1. Quantification of oligodendrocytes, microglia an androgen receptor at early time points after LPC injection. a**, Co-immunostaining of Iba-1 (microglia), PDGFRα (oligodendrocyte progenitors) and AR (androgen receptor) with CXCR4 (chemokine receptor) in the LPC demyelinated lesion at 10 days post-lesion. Adult castrated male mice received a subcutaneous (s.c.) Silastic implant, empty (LPC) or filled with testosterone (LPC+T). **b**. quantification of % of Iba-1 staining and number of PDGFRα and AR markers in LPC- and LPC+T-lesions. Cell nuclei were counterstained in blue with DAPI. Within the LPC lesion, delimited by the dotted line, Iba-1, PDGFRα, and AR were lowly immunostained in males receiving an empty implant (LPC), but highly immunostained in males receiving a T implant (LPC+T). Data are presented as means ± S.E.M. (two-tailed unpaired Student's t-test). **P<0.01, *P<0.05. Scale bar: 20 µm.

**
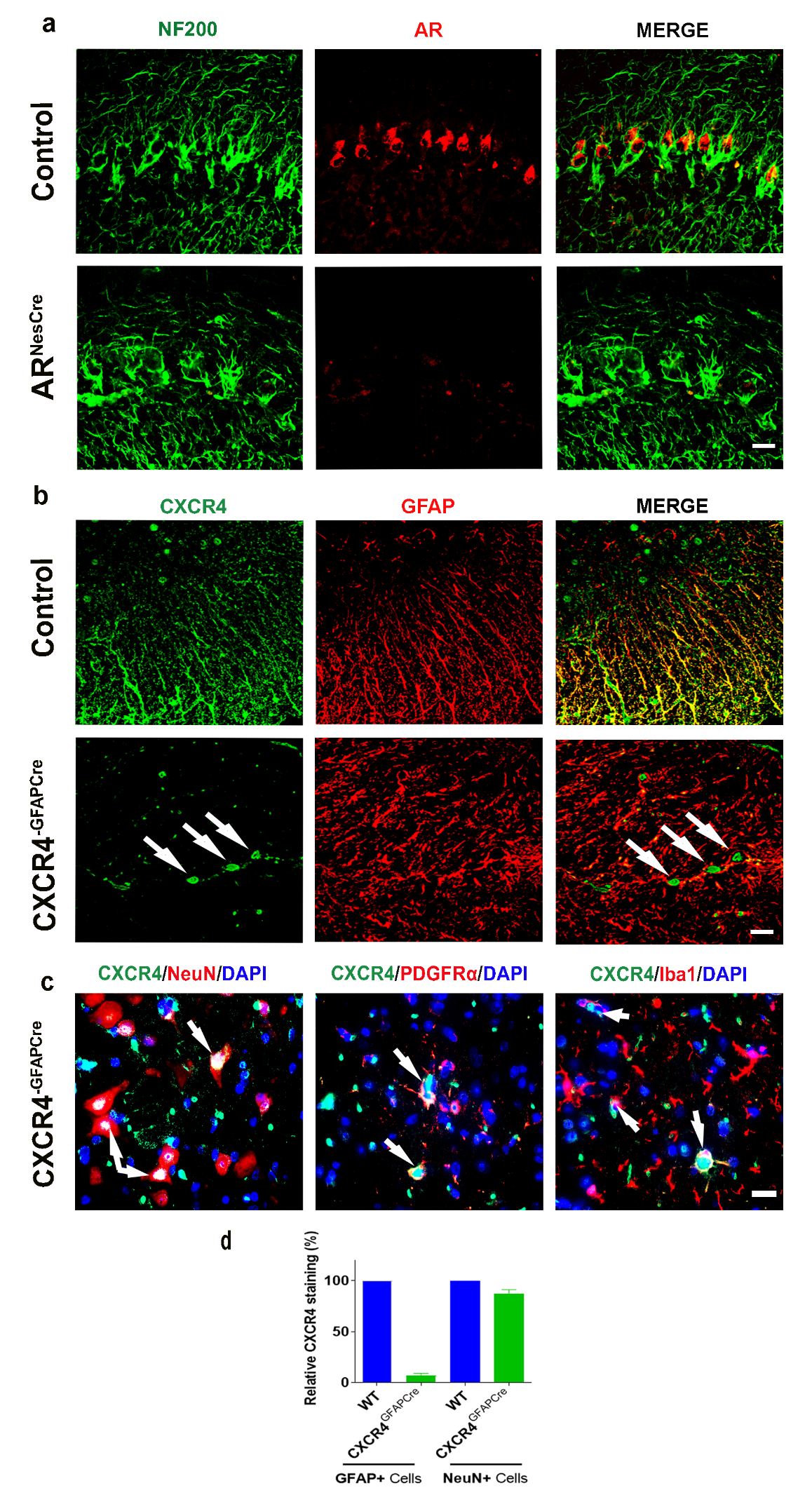
Supplemental Fig. 2. Validation of AR antibody specificity and astrocyte-specific genetic inactivation of CXCR4. a**, Double labeling of 200-kD neurofilament protein (NF200) and AR on cerebellar sections of wildtype (control: AR^lox/lox^) or AR^NesCre^ male mice with selective CNS ablation of AR. In the wildtype cerebellum, the monoclonal AR antibody stained the soma of Purkinje neurons. AR immunostaining was absent in cerebellar neurons of AR^NesCre^ mice. **b**, Double labeling of CXCR4 and GFAP on spinal cord white matter of CXCR4^Lox/Lox^ (control) or CXCR4^GFAPCre^ male mice with selective ablation of CXCR4 in astrocytes. The GFAP^+^ astrocytic processes of wildtype but not of CXCR4^GFAPCre^ mice were immunostained for CXCR4. Only vessels were stained for CXCR4 (arrows). **c**, Double immunolabeling of CXCR4/NeuN, CXCR4/PDGFRα and CXCR4/Iba1 in CXCR4^GFAPCre^ mice, indicating that CXCR4 continues to be expressed in neurons, OPC and microglia after its ablation in astrocytes. Scale bars = 20 µm in B and 10µm in **c**. **d**, Quantification of CXCR4 expression in GFAP^+^ astrocytes and NeuN^+^ neurons, showing a drastic decrease in CXCR4 expression in astrocytes and no decrease in neurons in the CXCR4^GFAPCre^ transgenic mice.

**
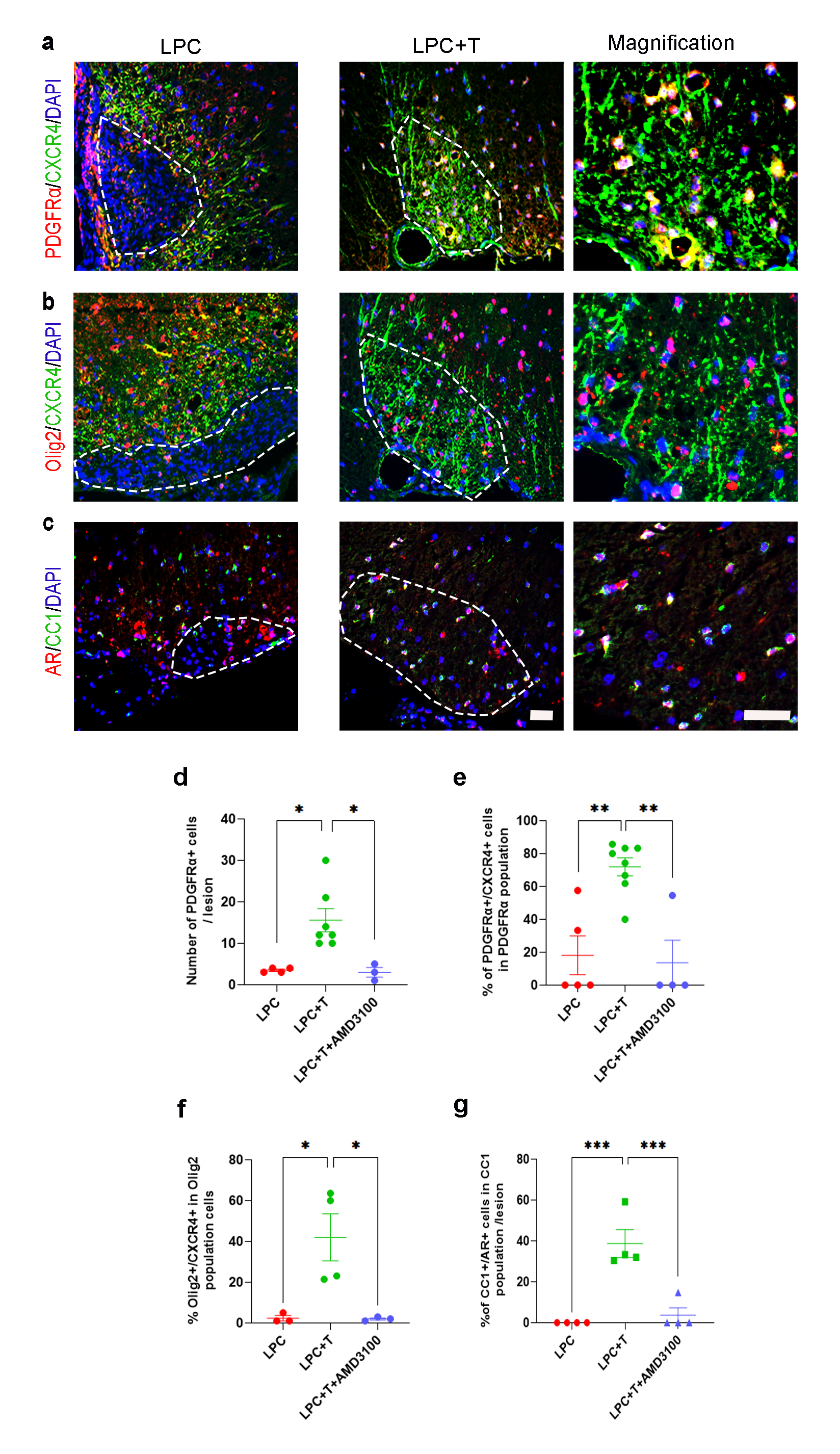
**

**Supplemental Fig. 3. Oligodendroglial cells express CXCR4 and AR. a**, Double labeling of the OPC marker PDGFRα and CXCR4 on spinal cord cross sections of castrated males at 30 dpl. Cell nuclei were counterstained in blue with DAPI. Within the LPC lesion, delimited by the dotted line, PDGFRα and CXCR4 co-staining were nearly absent in males receiving an empty implant (LPC), but were present (yellow stain) in males receiving a T implant (LPC+T). **d**,**e**, The T-dependent recruitment of PDGFRα^+^ and CXCR4^+^ cells was inhibited by AMD3100. **b**,**f**, Olig2^+^ oligodendroglial cells migrating into the LPC lesion of T-treated males also expressed CXCR4, and their appearance was blocked by AMD3100. **c**,**g**, CC1^+^ mature oligodendrocytes present in the LPC lesion of T-treated males expressed AR, and their appearance was blocked by AMD3100. Magnification of LPC+T panels show colocalization of CXCR4 with PDGFRα (top) and Olig2 (middle) and AR with CC1 (bottom). Data are presented as means ± S.E.M. (one-way ANOVA with Tukey's multiple comparisons tests). Asterisks mark significant differences. ^***^*P*<0.001, ^**^*P*<0.01, ^*^*P*<0.05. Scale bar: 20 µm.

**
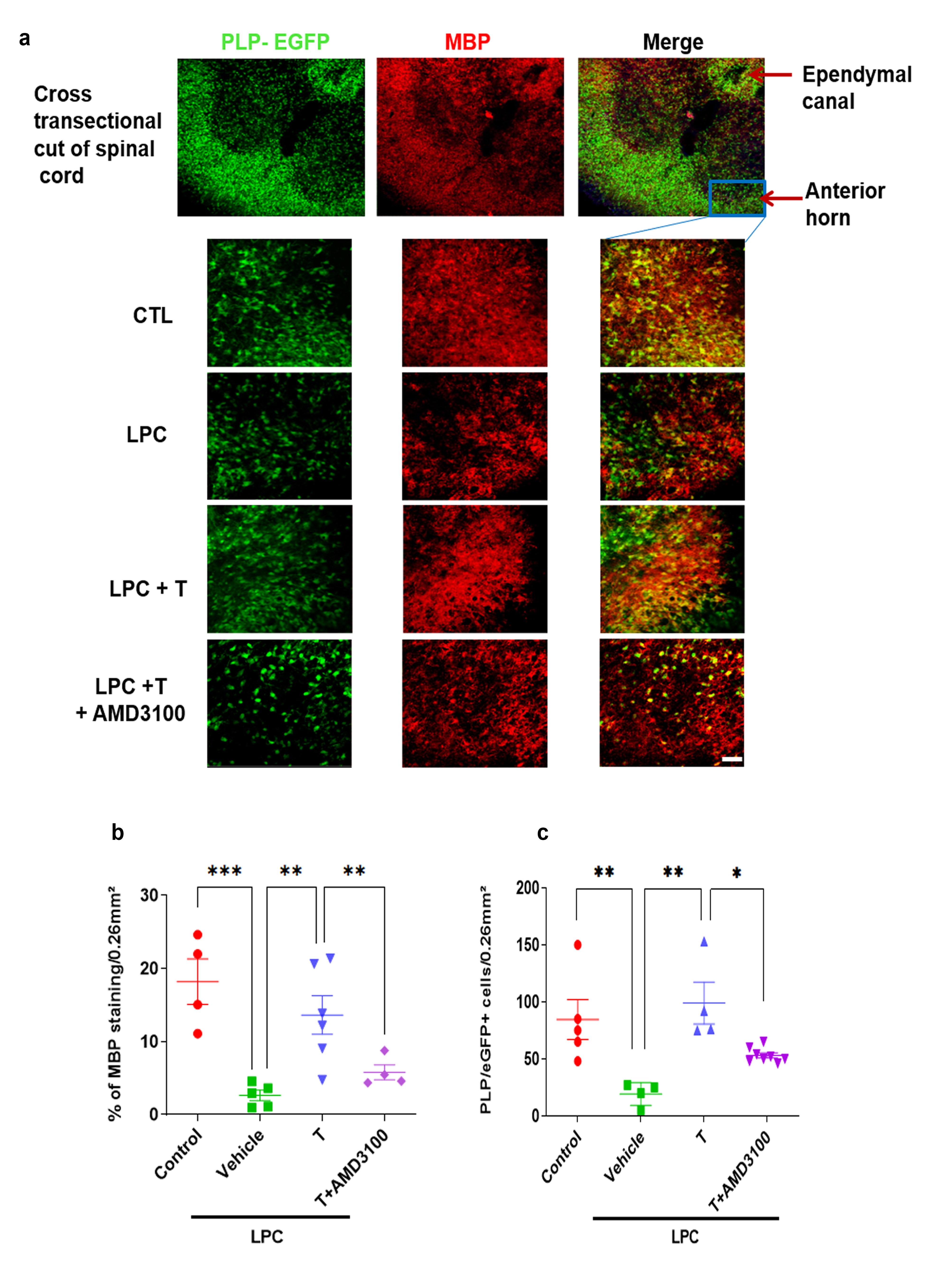
**

**Supplemental Fig. 4. Inhibition of CXCR4 blocks T-dependent remyelination in organotypic spinal cord cultures. a**, Organotypic cultures of coronal spinal cord slices from postnatal 10 (P10) PLP-eGFP mice. They were double-stained for eGFP (oligodendroglial cells) and MBP (CNS myelin). The blue square indicates the area analyzed for the different treatments. Control (CTL) slices were only exposed to vehicle (0.1% ethanol). Treated slices were exposed to 0.5 mg/ml LPC for 17-18 hrs to cause their demyelination. They were then treated with vehicle, T (1 µM) or T + AMD3100 (5 µM) during 5 days. The density of MBP immunostaining and of eGFP+ oligodendroglial cells were quantified. **a**,**b**, Slices treated with vehicle or AMD3100 alone remained largely demyelinated. T restored MBP^+^ myelin, and its remyelinating effect was inhibited by AMD3100. a,**c**, Slices treated with vehicle remained largely depleted of eGFP^+^ oligodendroglial cells. T treatment replenished eGFP^+^ cells, and its effect was again inhibited by AMD3100. Data are presented as mean ± S.E.M. (one-way ANOVA with Tukey's multiple comparisons tests). Asterisks mark significant differences. ^***^*P*<0.001, ^**^*P*<0.01, ^*^*P*<0.05. Scale bar: 20 µm.

**
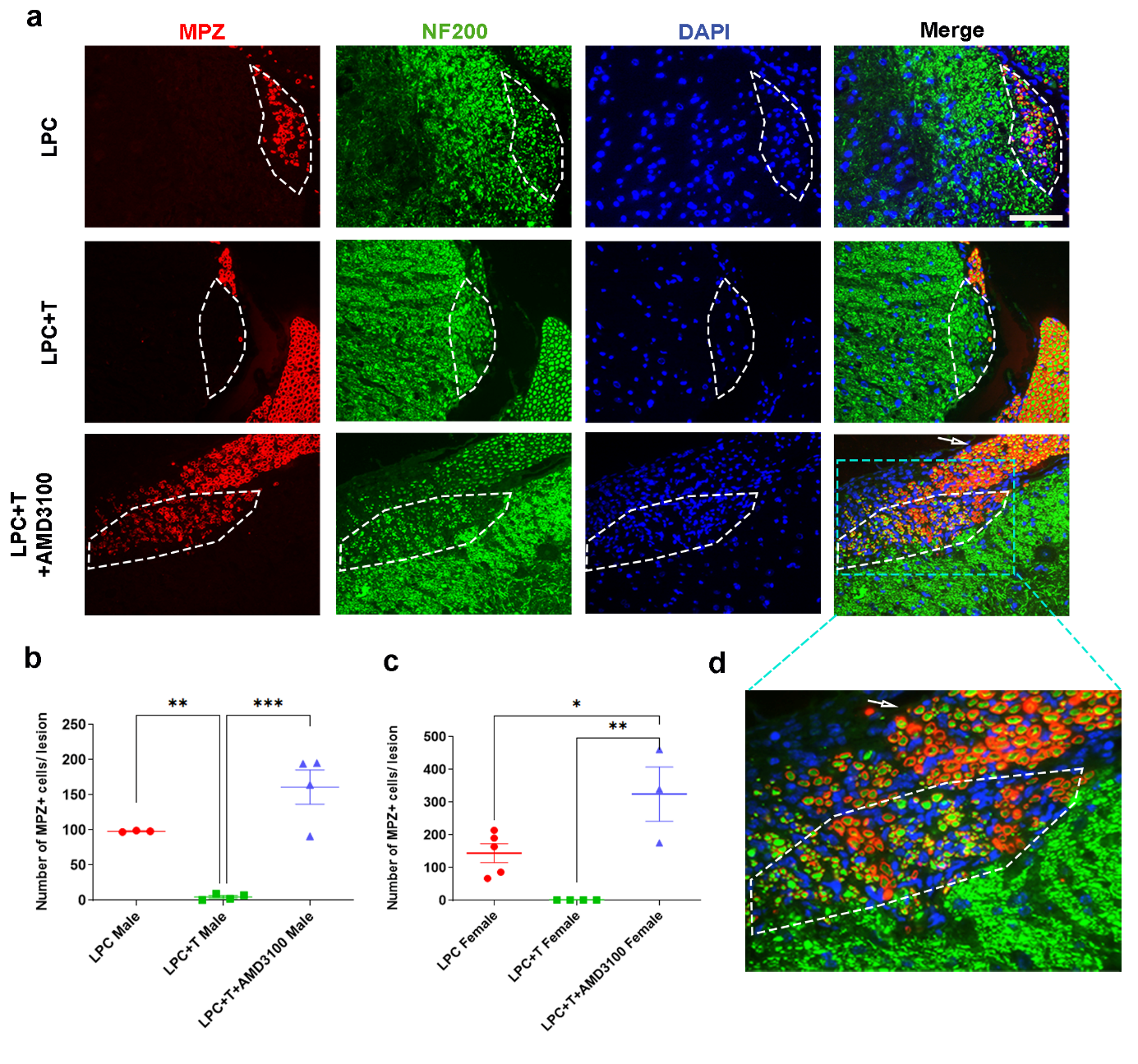
**

**Supplemental Fig. 5. Inhibition of CXCR4 promotes remyelination of the central nervous system by Schwann cells. a**, Double labeling of MPZ and NF200 on spinal cord cross sections of castrated males at 30 dpl. Cell nuclei were counterstained in blue with DAPI. Within the LPC lesion, delimited by a dotted line, no MPZ staining was observed in males receiving an implant filled with T, but MPZ^+^ Schwann cells migrated from ventral spinal roots (indicated by arrows) into the lesion in males treated with an empty implant or T+AMD3100 (Scale bar: 20 µm). **d**, Enlargement of the square (T+AMD3100 treatment) showed that several NF200^+^ axons within the lesion were remyelinated by MPZ^+^ myelin (Scale bar: 10 µm). **b**,**c**, In the LPC lesion, Schwann cells myelinated axons in the absence of T or CXCR4 signaling in both males and females. Data are presented as means ± S.E.M. (one-way ANOVA with Tukey's multiple comparisons tests). Asterisks mark significant differences. ^***^*P*<0.001, ^**^*P*<0.01, ^*^*P*<0.05.


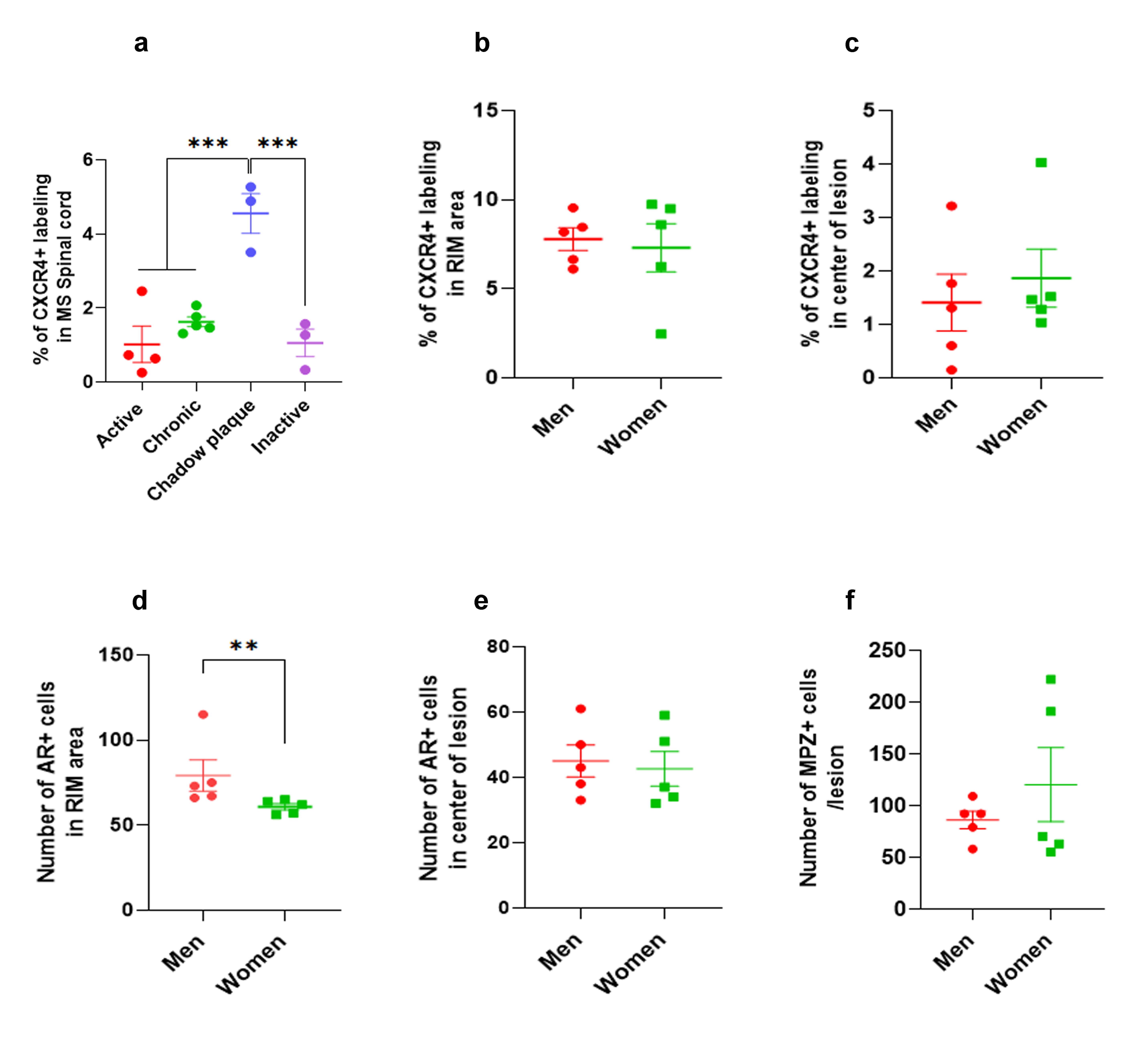


**Supplemental Fig. 6. Comparison of CXCR4, AR and MPZ immunostaining between men and women with MS**. **a**, Comparison of CXCR4 immunostaining between active, chronic, shadow and inactive MS lesions. **b**-**f**: Comparison of immunostaining between MS lesions in men (n = 5) and women (n = 5): (**b**) CXCR4 in RIM, (**c**) CXCR4 in lesion center, (**d**) number of AR^+^ cells in RIM, (**e**) number of AR^+^ cells in lesion center, (**f**) number of MPZ^+^ cells per lesion. Data are presented as means ± S.E.M. (one-way ANOVA with Tukey's multiple comparisons tests or two-tailed unpaired Student's t-test). Asterisks mark significant differences. ^**^*P*<0.01.

**Supplemental tables**

**Supplemental table 1:** **Representative differentially expressed genes in LPC+T- versus LPC-lesioned mice spinal cords.**

|  |  |  | **p-value** | **Fold-Change** |
| --- | --- | --- | --- | --- |
| **Transcript ID** | **Gene Symbol** | **RefSeq** | **LPC+T vs. LPC** | **LPC+T vs. LPC** |
| 17393868 | Mafb | NM_010658 | 0.0990157 | 2.00355 |
| 17487361 | Apoc2 | NM_001277944 | 0.0396738 | 2.01692 |
| 17288112 | 2010111I01Rik | NM_001289926 | 0.0727844 | 2.02149 |
| 17514687 | Phxr4 | NR_028271 | 0.0314935 | -2.02617 |
| 17357918 | Olfr1471 | NM_207132 | 0.0680273 | -2.03043 |
| 17222618 | Pantr1 | NR_027826 | 0.00614361 | -2.03651 |
| 17228576 | Fam20b | NM_145413 | 0.0246058 | -2.06919 |
| 17491026 | Emp3 | NM_001146346 | 0.0958955 | 2.07077 |
| 17242416 | D10Jhu81e | NM_138601 | 0.017692 | -2.07767 |
| 17360942 | Gal | NM_010253 | 0.249834 | 2.08394 |
| 17233226 | Lilrb4 | NM_001291894 | 0.235023 | 2.08623 |
| 17334166 | Dcpp1 | NM_019910 | 0.000200407 | 2.08652 |
| 17476273 | Zfp382 | NM_001081007 | 0.000818615 | 2.09772 |
| 17512611 | Dpep2 | NM_176913 | 0.0190516 | 2.09939 |
| 17226593 | Cxcr4 | NM_009911 | 0.0334029 | 2.11411 |
| 17229389 | Uck2 | XM_006497039 | 0.167014 | 2.12192 |
| 17437043 | Cytl1 | NM_001081106 | 0.0290707 | -2.12768 |
| 17332966 | Gm1604b | NM_001033442 | 0.0526153 | -2.13707 |
| 17340712 | Gm1604b | NM_001033442 | 0.0479483 | -2.15902 |
| 17494801 | Olfr493 | NM_146310 | 0.110178 | -2.16327 |
| 17423577 | Atp6v0d2 | NM_175406 | 0.0620034 | 2.19062 |
| 17340449 | Tfb1m | NM_146074 | 0.0176451 | 2.2199 |
| 17370437 | Dennd1a | ENSMUST00000132692 | 0.113863 | -2.22661 |
| 17362953 | Ms4a7 | NM_001025610 | 0.121279 | 2.22844 |
| 17233323 | Pln | NM_001141927 | 0.00191777 | -2.23738 |
| 17219418 | Cd84 | NM_001252472 | 0.137404 | 2.2471 |
| 17256784 | Tmem106a | NM_144830 | 0.0201331 | 2.35615 |
| 17238141 | Tac2 | NM_001199971 | 0.375016 | -2.39598 |
| 17291005 | Hist1h1b | NM_020034 | 0.136037 | 2.41828 |
| 17378741 | Nnat | NM_001291128 | 0.0239215 | -2.42416 |
| 17364098 | Acta2 | NM_007392 | 0.0294851 | -2.44142 |
| 17451614 | 2610524H06Rik | NM_181075 | 0.0116643 | -2.46748 |
| 17300247 | Trav9d-3 | ENSMUST00000103740 | 0.00712795 | -2.48699 |
| 17429279 | Olfr1337 | NM_146309 | 0.118667 | 2.55959 |
| 17230045 | Ifi204 | NM_008329 | 0.0128484 | 2.56509 |
| 17219222 | Mpz | NM_008623 | 0.0585987 | -2.57013 |
| 17291874 | Nrn1 | NM_153529 | 0.106129 | -2.57741 |
| 17267454 | Prr11 | NM_175563 | 0.0587645 | 2.60495 |
| 17533959 | Eif2s3x | XM_006528045 | 0.126424 | -2.6612 |
| 17287175 | Ogn | NM_008760 | 0.000154527 | -2.70873 |
| 17548411 | Hpse | AK202586 | 0.0645641 | 2.73784 |
| 17541776 | Cxx1a | NM_024170 | 0.103938 | 2.93492 |
| 17407412 | Sprr1a | NM_009264 | 0.0269404 | 2.96884 |
| 17458439 | Gpnmb | NM_053110 | 0.0835448 | 3.04123 |
| 17487570 | Vmn1r127 | NM_001166726 | 0.116983 | 3.20788 |
| 17333410 | Smok4a | NR_030763 | 0.0220034 | -3.35421 |
| 17469463 | Rybp | NM_019743 | 0.00603394 | -4.00471 |

*Data analysis of differentially expressed genes were conducted using the Partek Genomic Suite software (Partek Inc., St Louis, MO, USA). Those presenting a fold change superior or inferior of 2 were selected. N=4 animals for each group. Transcript ID, Gene symbols, Ref Seq, corrected p-values and fold changes in expression of studied genes*.

**Supplemental Table 2.** **Clinicopathologic Data on Multiple Sclerosis Patients and Controls**

2006-052

2007-085

2011-077

2013-019

2014-052

2008-072

2008-095

2011-048

2013-026

2014-072

69

66

66

53

61

57

61

53

54

54

7:30

6:30

9:35

7:15

10:00

7:55

9:15

10:00

6:40

7:55

6,14

6,18

6,45

6,81

6,88

6,50

6,88

6,38

6,54

6,60

F

F

F

F

F

M

M

M

M

M

PP

SP

PP

SP

SP

SP

SP

SP/PP?

SP

SP

Mean (F/M)

63/56

8:40/7:22

5/5

6,49/6,6

CODE

SEX

AGE (years)

PMD (h:min)

pH

MS Type

*F, female; M, male; MS, multiple sclerosis; PMD, postmortem delay (h: hours; min: minutes); PP primary progressive; SP secondary progressive.*

**Supplemental Table 3.** **Antibodies used for immunohistochemistry**

| **Antibody** | **Source** | **Identifier** |
| --- | --- | --- |
| Rabbit anti-Olig2  Mouse anti-Olig2  Rabbit anti-MBP  Mouse anti-MBP  Rat anti-MBP  Mouse anti- Adenomatous Polyposis Coli (CC1)  Mouse anti-Calbindin D-28K  Rabbit anti-Calbindin D-28K  Human CXCR4 (4G10)  Human/Mouse CXCL12/ SDF-1 MAb (Clone 79018) antibody  CD140a (PDGFRα) Monoclonal Antibody (APA5)  Glial Fibrillary Acidic Protein (Multipurpose) antibody  Mouse Anti-Glial Fibrillary Acidic Protein (GFAP) Monoclonal Antibody, Clone G-A-5  Androgen Receptor antibody (AR)  Mouse Anti-Neurofilament 200 kD (clone NE14)  P-Zero Myelin Protein (PZO) antibody (P0)  Anti IBA1 Polyclonal Antibody  Anti-mouse Alexa488 conjugated  Anti-mouse Alexa633 conjugated  Anti-rat Cy3 conjugated  Donkey anti-Rat IgG (H+L) Highly Cross-Adsorbed Secondary Antibody, Alexa Fluor™ 488 | Millipore  Millipore  Abcam  Millipore  Millipore  Millipore  Swant  Swant  Santa Cruz Biotechnology  R and D Systems  Thermo Fisher Scientific  Agilent  Sigma-Aldrich  Thermo Fisher Scientific  Millipore  Aves Labs  FUJIFILM WAKO  Thermo Fisher Scientific  Thermo Fisher Scientific  Jackson Immunoresearch  Thermo Fisher Scientific | Cat# AB9610; RRID: AB_570666  Cat# MABN50; RRID: AB_1141521  Cat# ab40390; RRID: AB_92396  Cat# MAB381; RRID: AB_11212910  Cat# MAB386; Clone 12; RRID: AB_94975  Cat# OP80; RRID: AB_2057371  Cat# 300; RRID: AB_10000340  Cat# CB-38; RRID: AB_10000347  Cat# sc-53534, RRID: AB_782002)  Cat# MAB350, RRID: AB_2088149  Cat# 16-1401-050, RRID: AB_2865868  Cat# Z0334, RRID: AB_10013382  Cat# G3893, RRID: AB_477010  Cat #MA5-13426, RRID: AB_11000751  Cat# MAB5256, RRID: AB_95184)  Cat# PZO, RRID: AB_2313561  Cat # 019-19741, RRID: AB_839504  Cat# A11070; RRID: AB_2534114  Cat# A-21053; RRID: AB_2535720  Cat# 111-165-144; RRID: AB_2338006  Cat# A-21208, RRID: AB_2535794 |
